# Cooperatively Catalyzed Activation of Thioglycosides with Iodine and Iron(III) Trifluoromethanesulfonate

**DOI:** 10.3390/molecules30153058

**Published:** 2025-07-22

**Authors:** Ashley R. Dent, Aidan M. DeSpain, Alexei V. Demchenko

**Affiliations:** Department of Chemistry, Saint Louis University, 3501 Laclede Ave, St. Louis, MO 63103, USA; ashley.dent@slu.edu (A.R.D.);

**Keywords:** glycosylation, ferric triflate, green chemistry, carbohydrates, thioglycosides

## Abstract

Reported herein is a further expansion of the cooperatively catalyzed Koenigs–Knorr glycosylation reaction, known as “the 4K reaction”. It has been discovered that molecular iodine, along with a metal salt and an acid additive, can activate thioglycosides. Previous mechanistic studies showed the interaction of the anomeric sulfur with thiophilic iodine; this complex is stable until the halophilic metal salt and the acid additive are added. This new avenue has allowed for the investigation of halophilic promoters that would not activate thioglycosides without iodine. Presented herein is the recent discovery of iron(III) triflate as an efficient activator of thioglycosides via the 4K reaction pathway.

## 1. Introduction

Carbohydrates are a vital class of molecules that are found profusely throughout nature. Due to their fundamental significance, it is of the utmost importance that carbohydrates be synthesized efficiently. The most central aspect to carbohydrate synthesis is the glycosylation reaction; however, it continues to be the most difficult and demanding aspect as well [1]. While nature effortlessly achieves glycosylation reactions via an enzymatic mechanism, chemical glycosylation itself remains onerous [2,3,4,5,6,7,8]. Paulsen’s statement written in 1982, “Although we have now learned to synthesize oligosaccharides, it should be emphasized that each oligosaccharide synthesis remains an independent problem, whose resolution requires considerable systematic research and a good deal of know-how. There are no universal reaction conditions for oligosaccharide syntheses” [2], is still current and very topical even now, many decades later – especially in relation to the synthesis of challenging linkages and targets. This article is dedicated to the late Professor Hans Paulsen (1922–2024), whose seminal publications on glycosylation, oligosaccharide synthesis, and building block reactivity have given enormous inspiration to the authors.

Early glycosylation reactions were performed by Michael [9], Fischer [10], and Koenigs/Knorr [11], making use of simple glycosyl halides or hemiacetals (Fischer). Although these simple glycosyl donors were appropriate for simple glycoside synthesis, the overall efficiency remained limited in scope and needed improvement. With classical Koenigs–Knorr reaction conditions [11,12,13], a glycosyl bromide donor is reacted with a glycosyl acceptor in the presence of silver(I) oxide or carbonate. Unfortunately, this reaction can be slow—particularly with unreactive bromides, such as those equipped with benzoyl protecting groups. Recently, we determined that a catalytic amount of a Lewis acid added to the Ag(I)-promoted glycosylation dramatically increases reaction rates and yields [14]. This has since been named “the 4K reaction” [15].

Thioglycosides are among the most common building blocks used in carbohydrate chemistry. For glycosylation, thioglycosides can be activated by the use of electrophilic or thiophilic promotors [16]. Among these, organosulfur compounds [17,18,19,20,21,22], halogen-based reagents [23,24,25,26,27,28], and photo-activators [29,30,31,32] are among the most popular. Activation with metal salts is also known [33]. Early work by Ferrier, who used mercury(II) salts for the activation of phenylthio glycosides [34], was complemented by Pohl, who used Ph_3_Bi(OTf)_2_ [35,36], and Sureshan [37] and Zhu [38], who used Au(III) salts. Our group previously reported the use of palladium(II) bromide [39], copper(II) bromide [40], and FeCl_3_ [41].

Our lab also recently demonstrated that thioglycosides can be activated using molecular iodine along with a metal salt and an acid additive under the 4K reaction conditions [42]. While initial studies were aimed at silver sulfate, a recent expansion explored bismuth(III) triflate as an activator while listing other viable metal salts [43]. Presented herein is the discovery that iron(III) triflate is also a viable reagent for the 4K reaction with thioglycosides.

## 2. Results and Discussion

**Figure 1:**
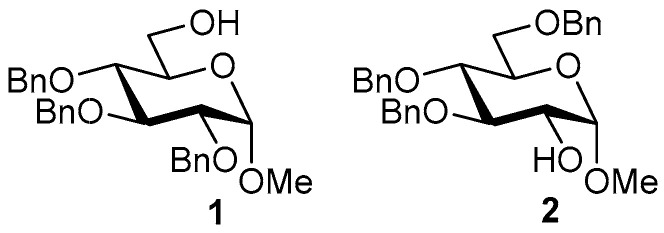
Structures of glycosyl acceptors **1** and **2** used in this study.

Ferric or iron(III) salts are formed from iron, the second most abundant metal on earth. Iron salts tend to be naturally abundant, inexpensive, and relatively benign. After preliminary optimization of the reaction conditions, we identified I_2_ (1.5 equiv), Fe(OTf)_3_ (1.5 equiv), and TfOH (0.2 equiv) in the presence of molecular sieves (3 Å) in 1,2-dichloroethane (DCE) as the most promising combination of reagents. I_2_ and Fe(OTf)_3_ without TfOH, or Fe(OTf)_3_ and TfOH without I_2_, will form the product too, but at much slower rates and lower yields. Reactions in the absence of Fe(OTf)_3_ did not proceed for unreactive thioglycosides. Scaling back the amounts of I_2_ and Fe(OTf)_3_ to 1.0 equiv led to a loss in yield and decrease in reaction rates. All glycosylations with less-reactive per-*O*-benzoylated (disarmed) glycosyl donors were performed at rt, whereas reactions with all other, more reactive glycosyl donors were performed at −30 °C. Standard primary glycosyl acceptor **1** and secondary glycosyl acceptor **2** (Figure 1) were chosen to investigate the scope of this reaction.

The results of glycosylation reactions are shown in Table 1. The glycosidation of per-*O*-benzoylated (disarmed) glucosyl donor **3** with 6-OH acceptor **1** produced disaccharide **4** in 16 h in 96% yield (entry 1). Glycosidation of **3** with 2-OH acceptor **2** produced disaccharide **5** in 24 h in 87% yield (entry 2). Other classes of thioglucosides were explored to further expand the scope. Thus, the glycosidation of α-ethylthio glycoside **6** with 6-OH acceptor **1** gave disaccharide **4** in 30 h in 92% yield (entry 3). The glycosidation of phenylthio or tolylthio glycosyl donors **7** or **8** with 6-OH acceptor **1** gave disaccharide **4** in 30 h in 90–95% yield (entries 4–5). These disaccharides all exhibit complete β-selectivity due to the participation of the neighboring benzoyl protecting group at C-2. The glycosidation of per-*O*-benzylated glucosyl donor **9** with 6-OH acceptor **1** was faster as these donors are known to be more reactive (armed) [44]. Disaccharide **10** was formed in 16 h in 86% yield (α:β = 1:1.5, entry 6). The glycosidation of **9** with 2-OH acceptor **2** formed disaccharide **11** in 16 h in 73% yield (α:β = 1.0:1, entry 7). Poor stereoselectivity was due to lack of the stereocontrolling factors.

We then looked into reactions of galactosyl donors. The glycosidation of per-*O*-benzoylated galactosyl donor **12** with 6-OH acceptor **1** produced disaccharide **13** in 16 h in 96% yield (entry 8). The glycosidation of **12** with 2-OH acceptor **2** produced disaccharide **14** in 24 h in 89% yield (entry 9). These disaccharides all exhibit complete β-selectivity due to the participation of the neighboring benzoyl protecting group at C-2. The glycosidation of per-*O*-benzylated galactosyl donor **15** with 6-OH acceptor **1** formed disaccharide **16** in 16 h in 92% yield (β-only, entry 10). The glycosidation of **15** with 2-OH acceptor **2** formed disaccharide **17** in 16 h in 79% yield (α:β = 1.6:1, entry 11). The poor stereoselectivity of **17** was due to the lack of stereocontrolling factors.

The glycosidation of per-*O*-benzoylated mannosyl donor **18** with 6-OH acceptor **1** produced disaccharide **19** in 84% yield in 24 h (entry 12). The glycosidation of **18** with 2-OH acceptor **2** produced disaccharide **20** in 24 h in 85% yield (entry 13). These disaccharides all exhibit complete α-selectivity due to the participation of the neighboring benzoyl protecting group at C-2. The glycosidation of per-*O*-benzylated mannosyl donor **21** with 6-OH acceptor **1** formed disaccharide **22** in 16 h in 81% yield (α:β = 1:4, entry 14). The glycosidation of **21** with 2-OH acceptor **2** formed disaccharide **23** in 16 h in 70% yield (α:β = 1:1.2, entry 15).

We then investigated whether these new reaction conditions would work well in combination with the remote benzoyl groups for galactosyl donors to achieve α-selectivity [45,46]. The glycosidation of galactosyl donor **24** with 6-OH acceptor **1** produced disaccharide **25** in 16 h in 78% yield with complete α-selectivity (entry 16). Finally, we investigated the application of this methodology to a mannosyl donor equipped with the superarming protecting group pattern [47]. The glycosidation of this mannosyl donor **26** with 6-OH acceptor **1** formed disaccharide **27** in 16 h in 79% yield with complete α-selectivity (entry 17).

Undoubtedly, our studies have demonstrated that I_2_/Fe(OTf)_3_/TfOH-catalyzed 4K reactions are swift and high yielding. Poor stereocontrol in the case of 2-*O*-benzylated glycosyl donors is not uncommon, and while α-galactosylation could be effectively achieved by using remote benzoyl groups (vide infra), we have not yet determined how to improve the stereoselectivity of glucosylation. To gain a stereocontrolling mode with glucosyl donors, in the past we demonstrated that bismuth(III)-promoted 4K reactions are compatible with the H-bond-mediated Aglycone Delivery (HAD) pathway [43]. The HAD reaction is based on glycosyl donors’ *O*-picoloyl (Pico) protecting group. These donors provide high or even complete *syn*-selectivities in respect to Pico [48]. The 4K reaction conditions developed herein, however, were found to be incompatible with 4-*O*-Pico-substituted building blocks. This is because ferric salts are known to be effective reagents for the removal of Pico groups [49].

## 3. Materials and Methods

### 3.1. General Methods

Column chromatography was performed on silica gel 60 (70–230 mesh); reactions were monitored by TLC on Kieselgel 60 F254. The compounds were detected by examination under UV light and by charring with 5% sulfuric acid in methanol. Solvents were removed under reduced pressure at <40 °C. ClCH_2_CH_2_Cl (1,2-DCE) was distilled from CaH_2_ directly prior to application. Molecular sieves (3 Å) used for reactions were first crushed and then activated in *vacuo* at 390 °C directly prior to application. Optical rotations were measured on a “Jasco P-2000” polarimeter (Jasco Corporation, Tokyo, Japan). ^1^H NMR spectra were recorded in CDCl_3_ at 400 or 700 MHz. ^13^C{^1^H} NMR spectra were recorded in CDCl_3_ at 101 or 175 MHz. The ^1^H NMR chemical shifts and the ^13^C{^1^H} NMR chemical shifts were referenced to CDCl_3_ (δ_H_ = 7.26, δ_C_ = 77.00 ppm). Structural assignments were made with additional information from COSY experiments. Compound ratios were determined by comparing the integration intensities of the relevant signals in their ^1^H NMR spectra. See Supplementary Materials for NMR spectra of all compounds. Accurate mass spectrometry determinations were performed using an Agilent 6230 ESI TOF LCMS mass spectrometer (Agilent, Santa Clara, CA, USA).

### 3.2. Synthesis of Building Blocks

**Methyl 2,3,4-tri-*O*-benzyl-α-D-glucopyranoside (1)** was synthesized as reported previously, and its analytical data was in accordance with that previously described [50].

**Methyl 3,4,6-tri-*O*-benzyl-α-D-glucopyranoside (2)** was synthesized as reported previously, and its analytical data was in accordance with that previously described [50].

**Ethyl 2,3,4,6-tetra-*O*-benzoyl-1-thio-β-D-glucopyranoside (3)** was synthesized as reported previously, and its analytical data was in accordance with that previously described [51].

**Ethyl 2,3,4,6-tetra-*O*-benzoyl-1-thio-α-D-glucopyranoside (6)** was synthesized as reported previously, and its analytical data was in accordance with that previously described [51].

**Phenyl 2,3,4,6-tetra-*O*-benzoyl-1-thio-β-D-glucopyranoside (7)** was synthesized as reported previously, and its analytical data was in accordance with that previously described [52].

**Tolyl 2,3,4,6-tetra-*O*-benzoyl-1-thio-β-D-glucopyranoside (8)** was synthesized as reported previously, and its analytical data was in accordance with that previously described [53].

**Ethyl 2,3,4,6-tetra-*O*-benzyl-1-thio-β-D-glucopyranoside (9)** was synthesized as reported previously, and its analytical data was in accordance with that previously described [54].

**Ethyl 2,3,4,6-tetra-*O*-benzoyl-1-thio-β-D-galactopyranoside (12)** was synthesized as reported previously, and its analytical data was in accordance with that previously described [51].

**Ethyl 2,3,4,6-tetra-*O*-benzyl-1-thio-β-D-galactopyranoside (15)** was synthesized as reported previously, and its analytical data was in accordance with that previously described [55].

**Ethyl 2,3,4,6-tetra-*O*-benzoyl-1-thio-β-D-mannopyranoside (18)** was synthesized as reported previously, and its analytical data was in accordance with that previously described [51].

**Ethyl 2,3,4,6-tetra-*O*-benzyl-1-thio-β-D-mannopyranoside (21)** was synthesized as reported previously, and its analytical data was in accordance with that previously described [56].

**Ethyl 3,4,-di-*O*-benzoyl-2,6-di-*O*-benzyl-1-thio-β-D-galactopyranoside (24)** was synthesized as reported previously, and its analytical data was in accordance with that previously described [45].

**Ethyl 2-*O*-benzoyl-3,4,6-tri-O-benzyl-1-thio-β-D-mannopyranoside (26)** was synthesized as reported previously, and its analytical data was in accordance with that previously described [57].

### 3.3. Synthesis of Disaccharides

**General procedure for glycosidation.** A mixture of glycosyl donor (0.05 mmol, 1.1 equiv), glycosyl acceptor (0.045 mmol, 1.0 equiv), and freshly activated molecular sieves (3 Å, 150 mg) in 1,2-dichloroethane (1.0 mL, 0.45 mM) was stirred under argon for 1 h at rt. I_2_ (0.0675 mmol, 1.5 equiv), Fe(OTf)_3_ (0.0675 mmol, 1.5 equiv), and TfOH (0.009 mmol, 0.2 equiv) were added, and the resulting mixture was stirred under argon at rt for the time specified in the tables and below. After that, the solids were filtered off through a pad of Celite and washed successively with CH_2_Cl_2_. The combined filtrate (~40 mL) was washed with 10% aq. Na_2_S_2_O_3_ (10 mL). The organic phase was separated, dried with sodium sulfate, and concentrated under reduced pressure. The residue was purified by column chromatography on silica gel (ethyl acetate—hexane or toluene gradient elution) to afford the respective disaccharides in yields and stereoselectivities listed in the tables and below. Anomeric ratios (or anomeric purity) were determined by comparison of the integral intensities of relevant signals in ^1^H NMR spectra.

**Methyl 6-*O*-(2,3,4,6-tetra-*O*-benzoyl-β-D-glucopyranosyl)-2,3,4-tri-*O*-benzyl-α-D-glucopyranoside (4)** was obtained from donor 3 [51] (39.0 mg, 0.06 mmol) and acceptor 1 [50] (25.7 mg, 0.055 mmol) under the general glycosidation method as a colorless foam in 16 h in 96% yield (55.4 mg, 0.053 mmol). The title compound was also obtained from donor 6 [51] (24.3 mg, 0.038 mmol) and acceptor 1 (16.0 mg, 0.034 mmol) under the general glycosidation method as a colorless foam in 30 h in 92% yield (32.8 mg, 0.031 mmol). The title compound was also obtained from donor 7 [52] (35.4 mg, 0.051 mmol) and acceptor 1 (21.7 mg, 0.047 mmol) under the general glycosidation method as a colorless foam in 30 h in 90% yield (43.9 mg, 0.042 mmol). The title compound was also obtained from donor 8 [53] (30.6 mg, 0.044 mmol) and acceptor 1 (18.4 mg, 0.04 mmol) under the general glycosidation method as a colorless foam in 30 h in 95% yield (39.1 mg, 0.037 mmol). Analytical data for 4 was in accordance with that previously reported [58].

**Methyl 2-*O*-(2,3,4,6-tetra-*O*-benzoyl-β-D-glucopyranosyl)-3,4,6-tri-*O*-benzyl-α-D-glucopyranoside (5)** was obtained from donor 3 [51] (34.0 mg, 0.053 mmol) and acceptor 2 [50] (22.4 mg, 0.048 mmol) under the general glycosidation method as a colorless form in 24 h in 87% yield (43.9 mg, 0.042 mmol). Analytical data for 5 was in accordance with that previously reported [50,58]

**Methyl 2,3,4-tri-*O*-benzyl-6-*O*-(2,3,4,6-tetra-*O*-benzyl-α/β-D-glucopyranosyl)-α-D-glucopyranoside (10)** was obtained from donor **9** [54] (24.4 mg, 0.042 mmol) and acceptor **1** [50] (17.6 mg, 0.038 mmol) under the general glycosidation method as a colorless form in 16 h in 86% yield (32.0 mg, 0.032 mmol). Analytical data for **10** was in accordance with that previously reported [59].

**Methyl 3,4,6-tri-*O*-benzyl-2-*O*-(2,3,4,6-tetra-*O*-benzyl-α/β-D-glucopyranosyl)-α-D-glucopyranoside (11)** was obtained from donor **9** [54] (24.8 mg, 0.042 mmol) and acceptor **2** [50] (17.9 mg, 0.039 mmol) under the general glycosidation method as a colorless form in 16 h in 73% yield (27.9 mg, 0.028 mmol). Analytical data for **11** was in accordance with that previously reported [59].

**Methyl 6-*O*-(2,3,4,6-tetra-*O*-benzoyl-β-D-galactopyranosyl)-2,3,4-tri-*O*-benzyl-α-D-glucopyranoside (13)** was obtained from donor 12 [51] (29.1 mg, 0.045 mmol) and acceptor 1 [50] (19.2 mg, 0.041 mmol) under the general glycosidation method as a colorless form in 16 h in 96% yield (41.4 mg, 0.040 mmol). Analytical data for 13 was in accordance with that previously reported [60].

**Methyl 2-*O*-(2,3,4,6-tetra-*O*-benzoyl-β-D-galactopyranosyl)-3,4,6-tri-*O*-benzyl-α-D-glucopyranoside (14)** was obtained from donor **12** [51] (24.4 mg, 0.038 mmol) and acceptor **2** [50] (16.1 mg, 0.035 mmol) under the general glycosidation method as a colorless form in 24 h in 89% yield (32.1 mg, 0.031 mmol). Analytical data for **14** was in accordance with that previously reported [61].

**Methyl 2,3,4-tri-*O*-benzyl-6-*O*-(2,3,4,6-tetra-*O*-benzyl-α/β-D-galactopyranosyl)-α-D-glucopyranoside (16)** was obtained from donor **15** [55] (22.3 mg, 0.038 mmol) and acceptor **1** [50] (16.1 mg, 0.035 mmol) under the general glycosidation method as a colorless foam in 16 h in 92% yield (31.5 mg, 0.032 mmol). Analytical data for **16** was in accordance with that previously reported [62].

**Methyl 3,4,6-tri-*O*-benzyl-2-*O*-(2,3,4,6-tetra-*O*-benzyl-α/β-D-galactopyranosyl)-α-D-glucopyranoside (17)** was obtained from donor **15** [55] (22.3 mg, 0.038 mmol) and acceptor **2** [50] (16.1 mg, 0.035 mmol) under the general glycosidation method as a colorless form in 16 h in 79% yield (27.1 mg, 0.027 mmol). Analytical data for **17** was in accordance with that previously reported [63].

**Methyl 6-*O*-(2,3,4,6-tetra-*O*-benzoyl-α-D-mannopyranosyl)-2,3,4-tri-*O*-benzyl-α-D-glucopyranoside (19)** was obtained from donor **18** [51] (24.4 mg, 0.038 mmol) and acceptor **1** [50] (16.1 mg, 0.035 mmol) under the general glycosidation method as a colorless foam in 24 h in 84% yield (30.1 mg, 0.029 mmol). Analytical data for **19** was in accordance with that previously reported [64].

**Methyl 2-*O*-(2,3,4,6-tetra-*O*-benzoyl-α-D-mannopyranosyl)-3,4,6-tri-*O*-benzyl-α-D-glucopyranoside (20)** was obtained from donor **18** [51] (21.6 mg, 0.034 mmol) and acceptor **2** [50] (14.2 mg, 0.031 mmol) under the general glycosidation method as a colorless form in 24 h in 85% yield (26.9 mg, 0.026 mmol). Analytical data for **20** was in accordance with that previously reported [65].

**Methyl 2,3,4-tri-*O*-benzyl-6-*O*-(2,3,4,6-tetra-*O*-benzyl-α/β-D-mannopyranosyl)-α-D-glucopyranoside (22)** was obtained from donor **21** [56] (30.4 mg, 0.052 mmol) and acceptor **1** [50] (21.9 mg, 0.047 mmol) under the general glycosidation method as a colorless foam in 16 h in 81% yield (37.5 mg, 0.038 mmol). Analytical data for **22** was in accordance with that previously reported [66].

**Methyl 3,4,6-tri-*O*-benzyl-2-*O*-(2,3,4,6-tetra-*O*-benzyl-α/β-D-mannopyranosyl)-α-D-glucopyranoside (23)** was obtained from donor **21** [56] (30.5 mg, 0.052 mmol) and acceptor **2** [50] (22.0 mg, 0.047 mmol) under the general glycosidation method as a colorless form in 16 h in 70% yield (32.9 mg, 0.033 mmol). Analytical data for **23** was in accordance with that previously reported [67].

**Methyl 6-*O*-(3,4-di-*O*-benzoyl-2,6-di-*O*-benzyl-α-D-galactopyranosyl)-2,3,4-tri-*O*-benzyl-α-D-glucopyranoside (25)** was obtained from donor 24 [45] (27.9 mg, 0.046 mmol) and acceptor 1 [50] (19.2 mg, 0.041 mmol) under the general glycosidation method as a colorless foam in 16 h in 78% yield (32.5 mg, 0.032 mmol). Analytical data for 25 was in accordance with that previously reported [45].

**Methyl 6-*O*-(2-*O*-benzoyl-3,4,6-tri-*O*-benzyl-α-D-mannopyranosyl)-2,3,4-tri-*O*-benzyl-α-D-glucopyranoside (27)** was obtained from donor 26 [57] (30.1 mg, 0.05 mmol) and acceptor 1 [50] (21.2 mg, 0.046 mmol) under the general glycosidation method as a colorless foam in 16 h in 79% yield (36.2 mg, 0.036 mmol). Analytical data for 27 was in accordance with that previously reported. [68]

## 4. Conclusions

Our previous studies of the 4K reaction with thioglycosides opened a new exciting avenue for discovery of new classes of thioglycoside activators. Previously, we demonstrated that I_2_/Bi(OTf)_3_/TfOH cooperatively catalyzed 4K reactions can be swift and efficient. Developed herein is an I_2_/Fe(OTf)_3_/TfOH cooperatively catalyzed 4K reaction for the direct activation of conventional thioglycosides. This methodology presents a promising expansion of the 4K reaction, which overall continues to be an exciting avenue for exploration. As demonstrated by several substrates and targets, this method offers new synthetic capabilities. The reaction conditions are relatively mild, and because of that, the glycosylation reactions are much slower than those in the presence of bismuth(III), albeit the product yields were similar.

Regardless of whether bismuth(III) or iron(III) were employed as co-catalysis, the reaction worked with a broad range of substrates, with both armed and disarmed glycosyl donors. In contrast, previously described activations in the presence of FeCl_3_ alone worked well only for highly reactive, armed and superarmed thioglycoside donors, even in the presence of a large excess. Attempts to substitute Fe(OTf)_3_ with FeCl_3_ in the 4K reactions lead to decreased yields due to the accumulation of multiple side products.

It also became apparent that the 4K reactions with ferric triflate are not compatible with the HAD method because ferric salts are known to cleave the picoloyl group that is needed as a stereodirecting handle. Further exploration of the 4K reaction, the search for other effective promoters and catalysts, and application to the manual and automated synthesis of various linkages and glycan targets are currently underway.

## Figures and Tables

**Table 1:** Investigation of the scope of the iron(III) triflate-promoted 4K reaction.

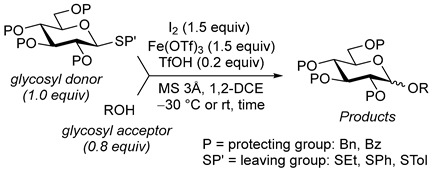			
Entry	Donor	Acceptor	Product, Time, Yield, Ratio α/β
1	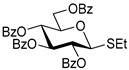 **3**	**1**	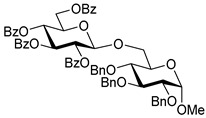 **4**, 16 h, 96%, β-only
2	**3**	**2**	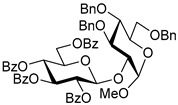 **5**, 24 h, 87%, β-only
3	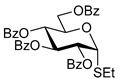 **6**	**1**	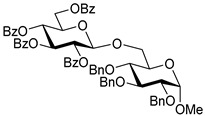 **4**, 30 h, 92%, β-only
4	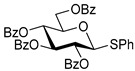 **7**	**1**	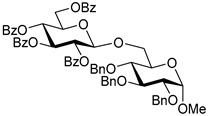 **4**, 30 h, 90%, β-only
5	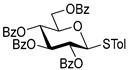 **8**	**1**	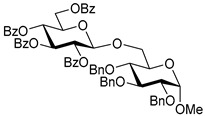 **4**, 30 h, 95%, β-only
6	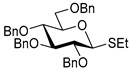 **9**	**1**	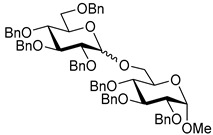 **10**, 16 h, 86%, α:β = 1:1.5
7	**9**	**2**	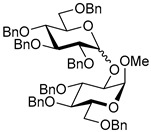 **11**, 16 h, 73%, α:β = 1:1
8	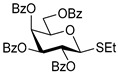 **12**	**1**	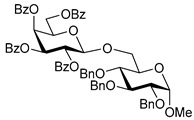 **13**, 16 h, 96%, β-only
9	**12**	**2**	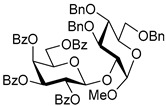 **14**, 24 h, 89%, β-only
10	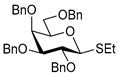 **15**	**1**	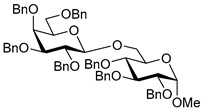 **16**, 16 h, 92%, β-only
11	**15**	**2**	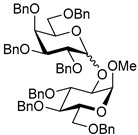 **17**, 16 h, 79%, α:β = 1.6:1
12	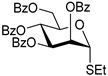 **18**	**1**	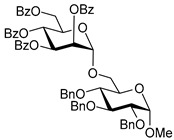 **19**, 24 h, 84%, α-only
13	**18**	**2**	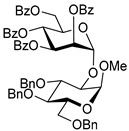 **20**, 24 h, 85%, α-only
14	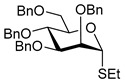 **21**	**1**	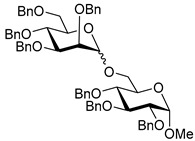 **22**, 16 h, 81%, α:β = 1:4
15	**21**	**2**	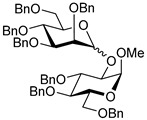 **23**, 16 h, 70%, α:β = 1:1.2
16	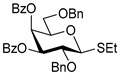 **24**	**1**	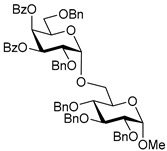 **25**, 16 h, 78%, α-only
17	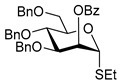 **26**	**1**	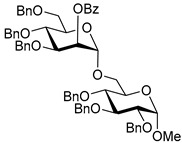 **27**, 16 h, 79%, α-only

## Data Availability

All data is available from the authors.

## Supplementary Materials

The following supporting information can be downloaded at: https://www.mdpi.com/article/10.3390/molecules30153058/s1, Figure S1. ^1^H NMR Spectrum (CDCl_3_, 400 MHz) of Compound **4**; Figure S2. COSY NMR Spectrum (CDCl_3_, 400 MHz) of Compound **4**; Figure S3. ^13^C NMR Spectrum (CDCl_3_, 101 MHz) of Compound **4**; Figure S4. ^1^H NMR Spectrum (CDCl_3_, 400 MHz) of Compound **5**; Figure S5. COSY NMR Spectrum (CDCl_3_, 400 MHz) of Compound **5**; Figure S6. ^13^C NMR Spectrum (CDCl_3_, 101 MHz) of Compound **5**; Figure S7. ^1^H NMR Spectrum (CDCl_3_, 400 MHz) of Compound **10;** Figure S8. COSY NMR Spectrum (CDCl_3_, 400 MHz) of Compound **10**; Figure S9. ^13^C NMR Spectrum (CDCl_3_, 101 MHz) of Compound **10**; Figure S10. ^1^H NMR Spectrum (CDCl_3_, 400 MHz) of Compound **11**; Figure S11. COSY NMR Spectrum (CDCl_3_, 400 MHz) of Compound **11**; Figure S12. ^13^C NMR Spectrum (CDCl_3_, 101 MHz) of Compound **11**; Figure S13. ^1^H NMR Spectrum (CDCl_3_, 400 MHz) of Compound **13**; Figure S14. COSY NMR Spectrum (CDCl_3_, 400 MHz) of Compound **13**; Figure S15. ^13^C NMR Spectrum (CDCl_3_, 101 MHz) of Compound **13**; Figure S16. ^1^H NMR Spectrum (CDCl_3_, 400 MHz) of Compound **14**; Figure S17. COSY NMR Spectrum (CDCl_3_, 400 MHz) of Compound **14**; Figure S18. ^13^C NMR Spectrum (CDCl_3_, 101 MHz) of Compound **14**; Figure S19. ^1^H NMR Spectrum (CDCl_3_, 400 MHz) of Compound **16**; Figure S20. COSY NMR Spectrum (CDCl_3_, 400 MHz) of Compound **16**; Figure S21. ^13^C NMR Spectrum (CDCl_3_, 101 MHz) of Compound **16**; Figure S22. ^1^H NMR Spectrum (CDCl_3_, 400 MHz) of Compound **17**; Figure S23. COSY NMR Spectrum (CDCl_3_, 400 MHz) of Compound **17**; Figure S24. ^13^C NMR Spectrum (CDCl_3_, 101 MHz) of Compound **17**; Figure S25. ^1^H NMR Spectrum (CDCl_3_, 400 MHz) of Compound **19**; Figure S26. COSY NMR Spectrum (CDCl_3_, 400 MHz) of Compound **19**; Figure S27. ^13^C NMR Spectrum (CDCl_3_, 101 MHz) of Compound **19**; Figure S28. ^1^H NMR Spectrum (CDCl_3_, 400 MHz) of Compound **20**; Figure S29. COSY NMR Spectrum (CDCl_3_, 400 MHz) of Compound **20**; Figure S30. ^13^C NMR Spectrum (CDCl_3_, 101 MHz) of Compound **20**; Figure S31. ^1^H NMR Spectrum (CDCl_3_, 400 MHz) of Compound **22**; Figure S32. COSY NMR Spectrum (CDCl_3_, 400 MHz) of Compound **22**; Figure S33. ^13^C NMR Spectrum (CDCl_3_, 101 MHz) of Compound **22**; Figure S34. ^1^H NMR Spectrum (CDCl_3_, 400 MHz) of Compound **23**; Figure S35. COSY NMR Spectrum (CDCl_3_, 400 MHz) of Compound **23**; Figure S36. ^13^C NMR Spectrum (CDCl_3_, 101 MHz) of Compound **23**; Figure S37. ^1^H NMR Spectrum (CDCl_3_, 400 MHz) of Compound **25**; Figure S38. COSY NMR Spectrum (CDCl_3_, 400 MHz) of Compound **25**; Figure S39. ^13^C NMR Spectrum (CDCl_3_, 101 MHz) of Compound **25**; Figure S40. ^1^H NMR Spectrum (CDCl_3_, 400 MHz) of Compound **27**; Figure S41. COSY NMR Spectrum (CDCl_3_, 400 MHz) of Compound **27**; Figure S42. ^13^C NMR Spectrum (CDCl_3_, 101 MHz) of Compound **27**.
