# Supplementary material for: Cooperatively Catalyzed Activation of Thioglycosides with Iodine and Iron(III) Trifluoromethanesulfonate
Source: Molecules. 2025 Jul 22;30(15):3058. doi: 10.3390/molecules30153058 (PMC12348131; doi:10.3390/molecules30153058)
Supplement: Supplementary file 1 [file molecules-30-03058-s001.zip › molecules-3730252-supplementary.pdf]

Cooperatively Catalyzed Activation of Thioglycosides in the presence of Iron(III) Trifluoromethanesulfonate

Ashley R. Dent, Aidan M. DeSpain, and Alexei V. Demchenko*

Department of Chemistry, Saint Louis University, 3501 Laclede Ave, St. Louis, MO 63103, USA; e-mail: alexei.demchenko@slu.edu

NMR spectra

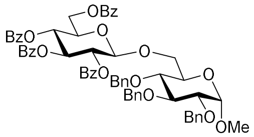

Methyl 6-*O*-(2,3,4,6-tetra-*O*-benzoyl-β-D-glucopyranosyl)-2,3,4-tri-*O*-benzyl-*α*-D-glucopyranoside (4)

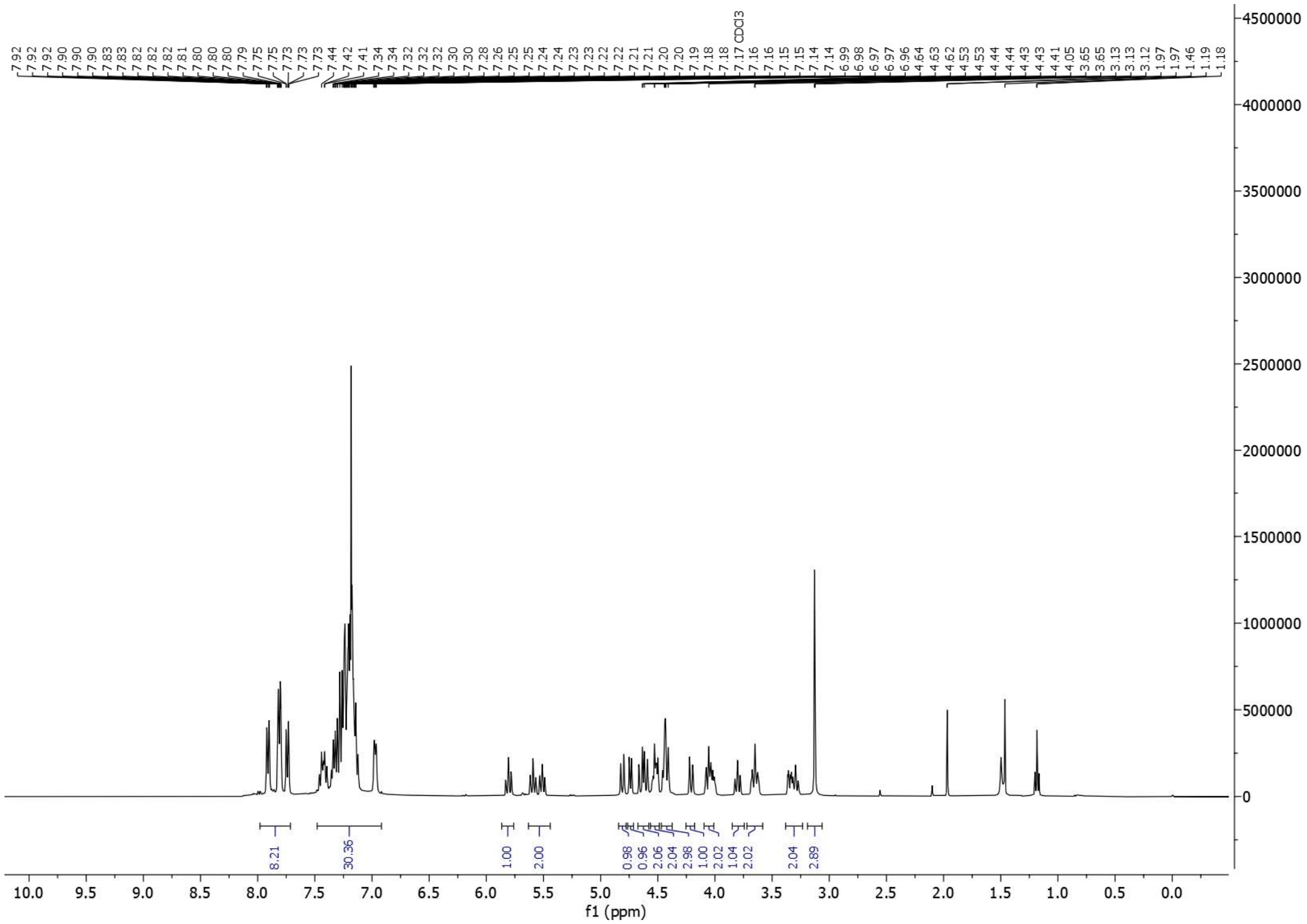

Figure S1. ^{1}H NMR Spectrum ($CDCl_3$, 400 MHz) of Compound **4.**

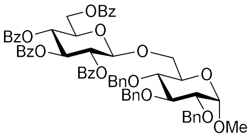

Methyl 6-*O*-(2,3,4,6-tetra-*O*-benzoyl-β-D-glucopyranosyl)-2,3,4-tri-*O*-benzyl-α-D-glucopyranoside (4)

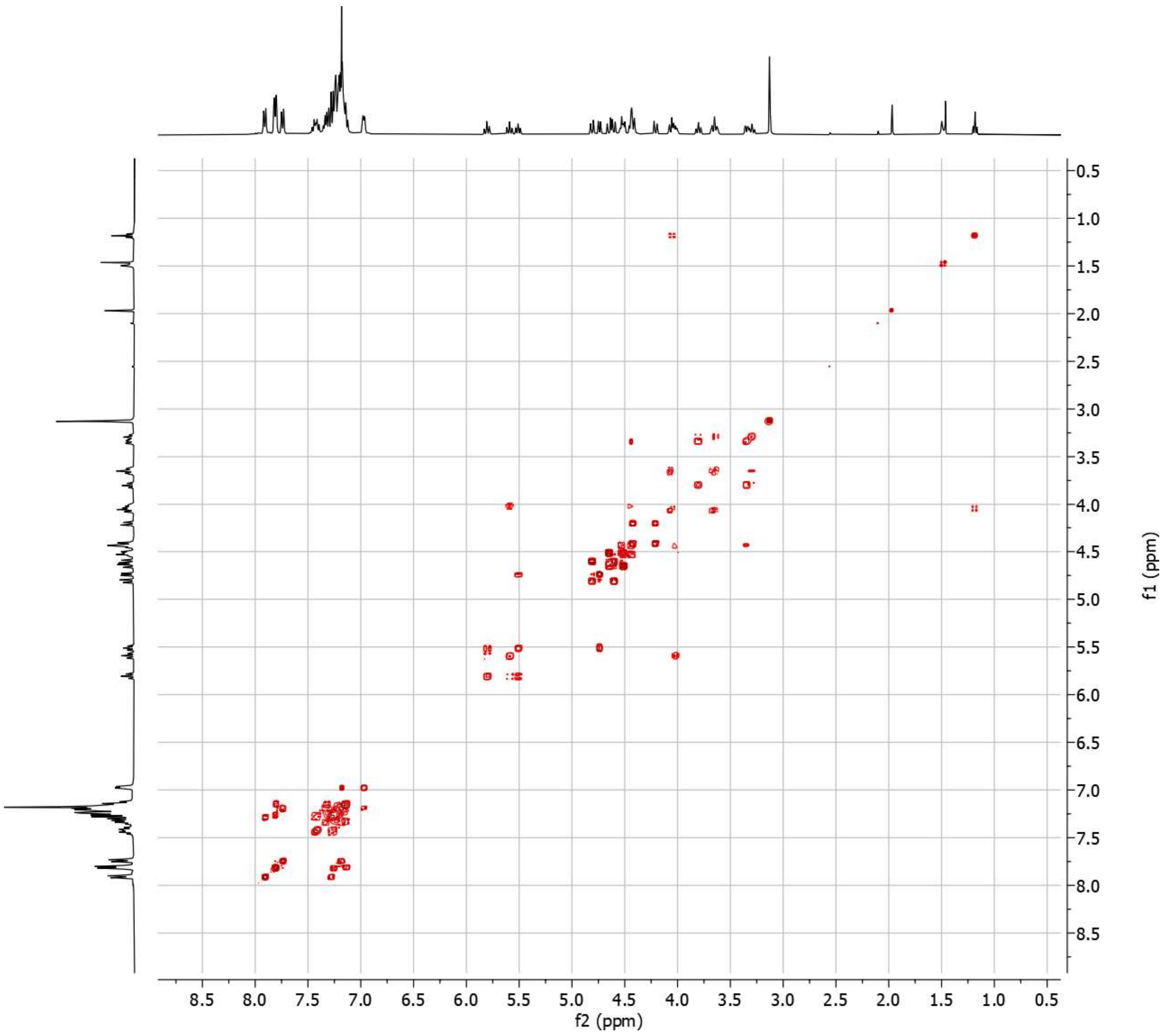

Figure S2. COSY NMR Spectrum ($CDCl_3$, 400 MHz) of Compound **4**.

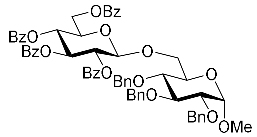

Methyl 6-*O*-(2,3,4,6-tetra-*O*-benzoyl-β-D-glucopyranosyl)-2,3,4-tri-*O*-benzyl-α-D-glucopyranoside (4)

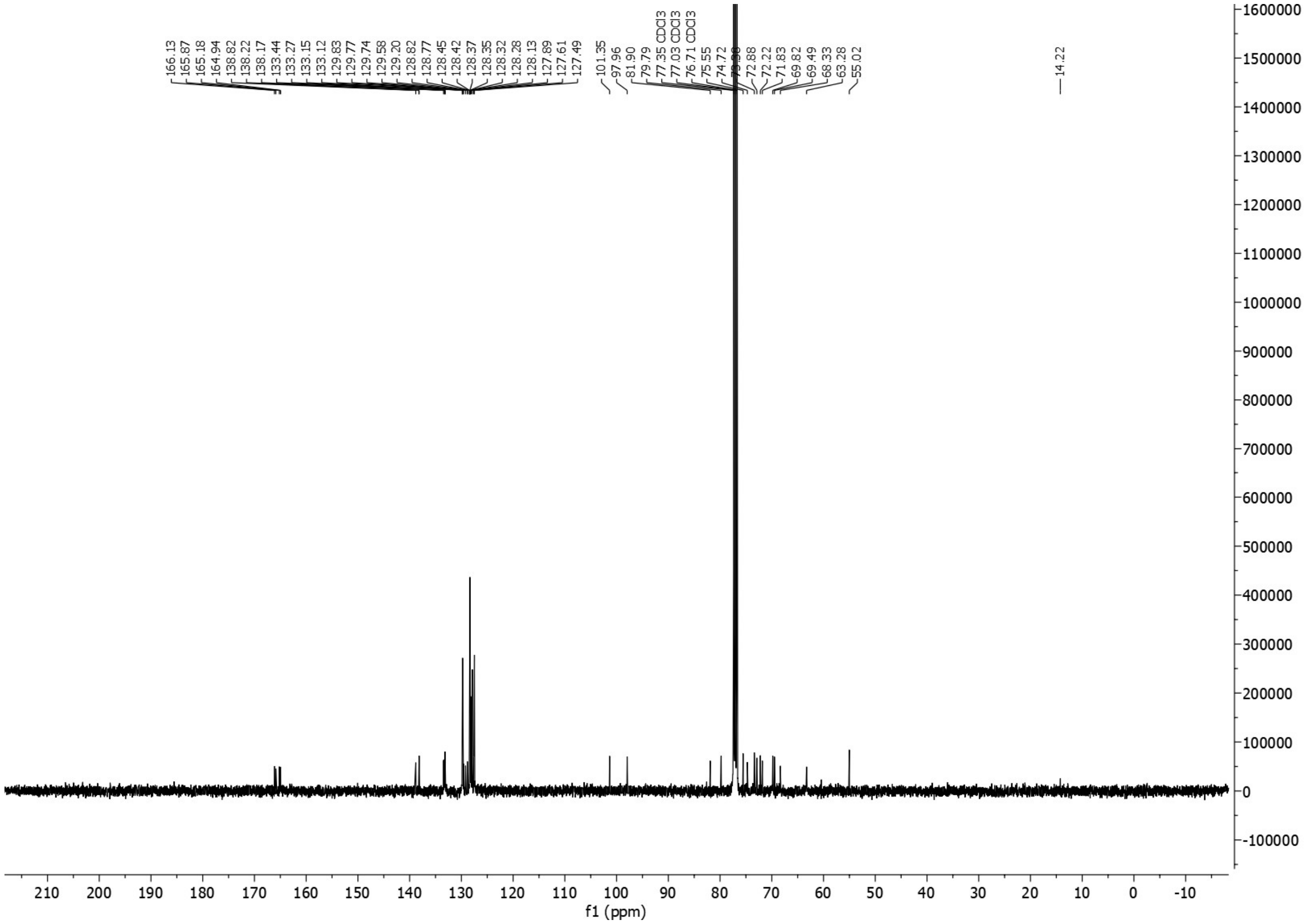

Figure S3. ^{13}C NMR Spectrum ($CDCl_3$, 101 MHz) of Compound **4**.

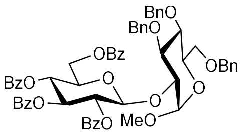

Methyl 2-*O*-(2,3,4,6-tetra-*O*-benzoyl-β-D-glucopyranosyl)-3,4,6-tri-*O*-benzyl-α-D-glucopyranoside (5)

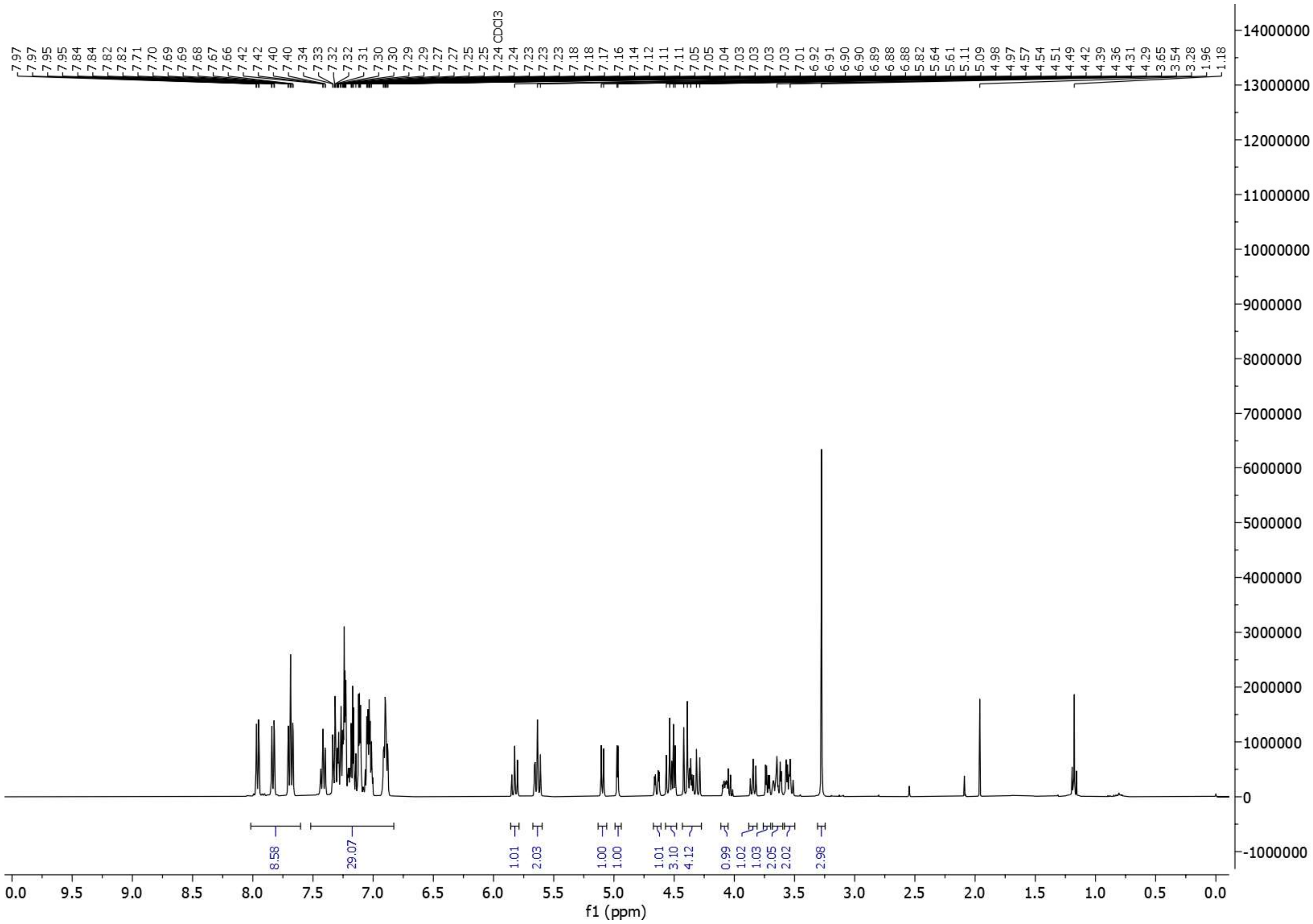

Figure S4. ^{1}H NMR Spectrum ($CDCl_3$, 400 MHz) of Compound **5.**

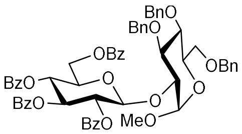

Methyl 2-*O*-(2,3,4,6-tetra-*O*-benzoyl-β-D-glucopyranosyl)-3,4,6-tri-*O*-benzyl-α-D-glucopyranoside (5)

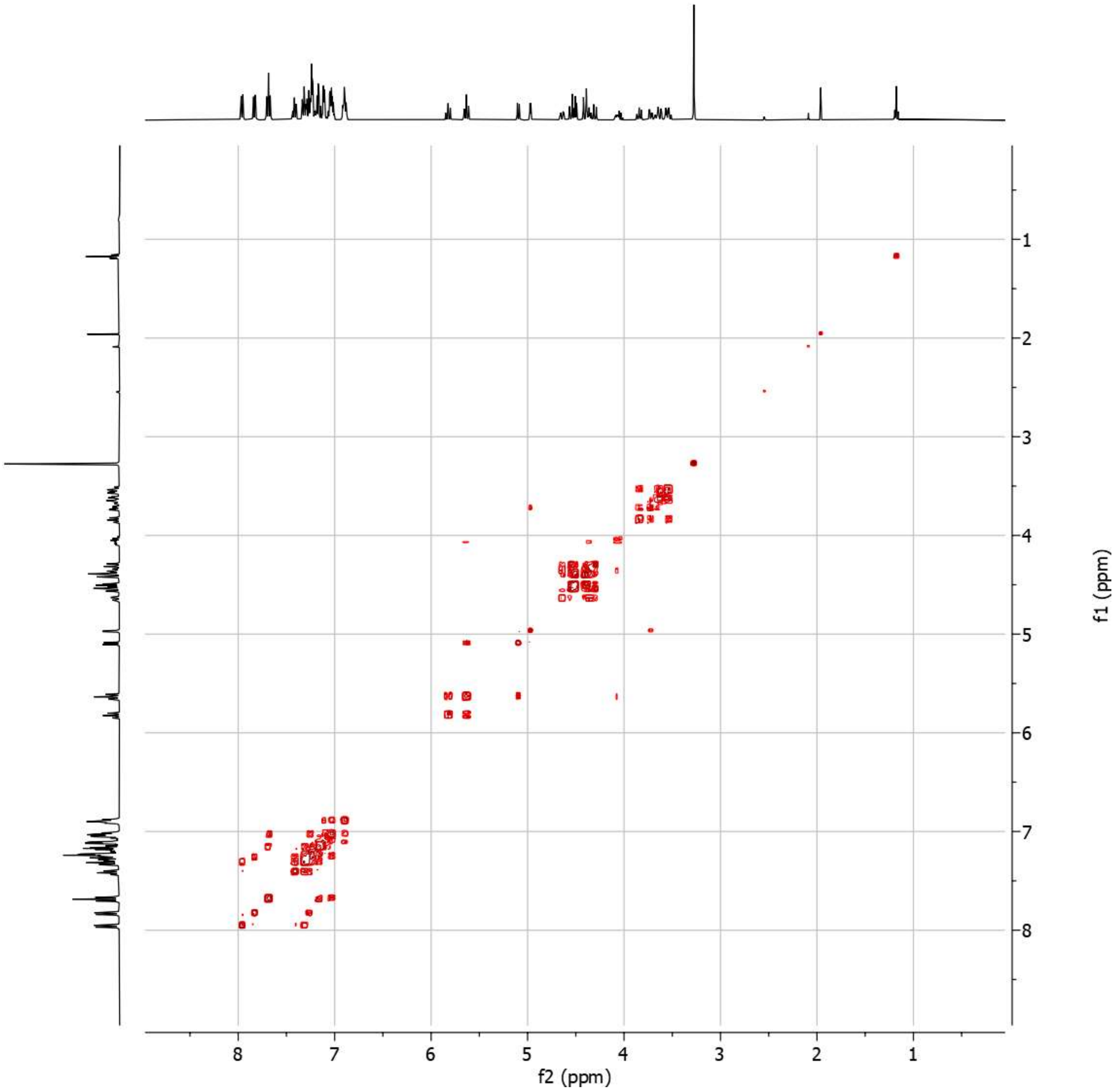

Figure S5. COSY NMR Spectrum ($CDCl_3$, 400 MHz) of Compound **5**.

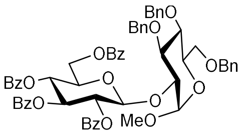

Methyl 2-*O*-(2,3,4,6-tetra-*O*-benzoyl-β-D-glucopyranosyl)-3,4,6-tri-*O*-benzyl-*α*-D-glucopyranoside (5)

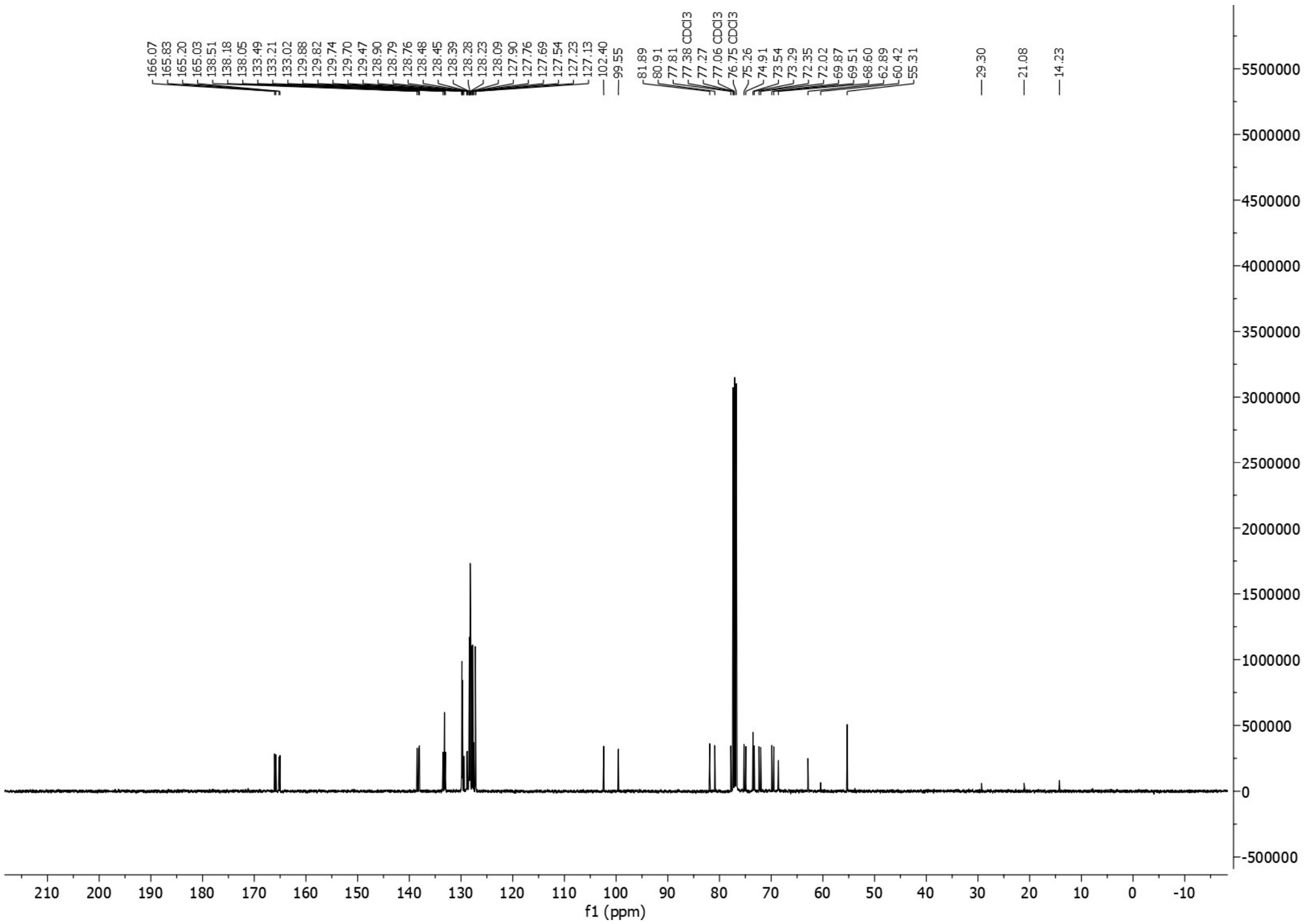

Figure S6. ^{13}C NMR Spectrum ($CDCl_3$, 101 MHz) of Compound **5**.

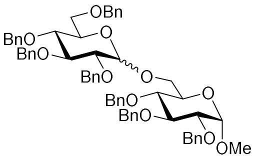

Methyl 2,3,4-tri-*O*-benzyl-6-*O*-(2,3,4,6-tetra-*O*-benzyl-α/β-D-glucopyranosyl)-α-D-glucopyranoside (10)

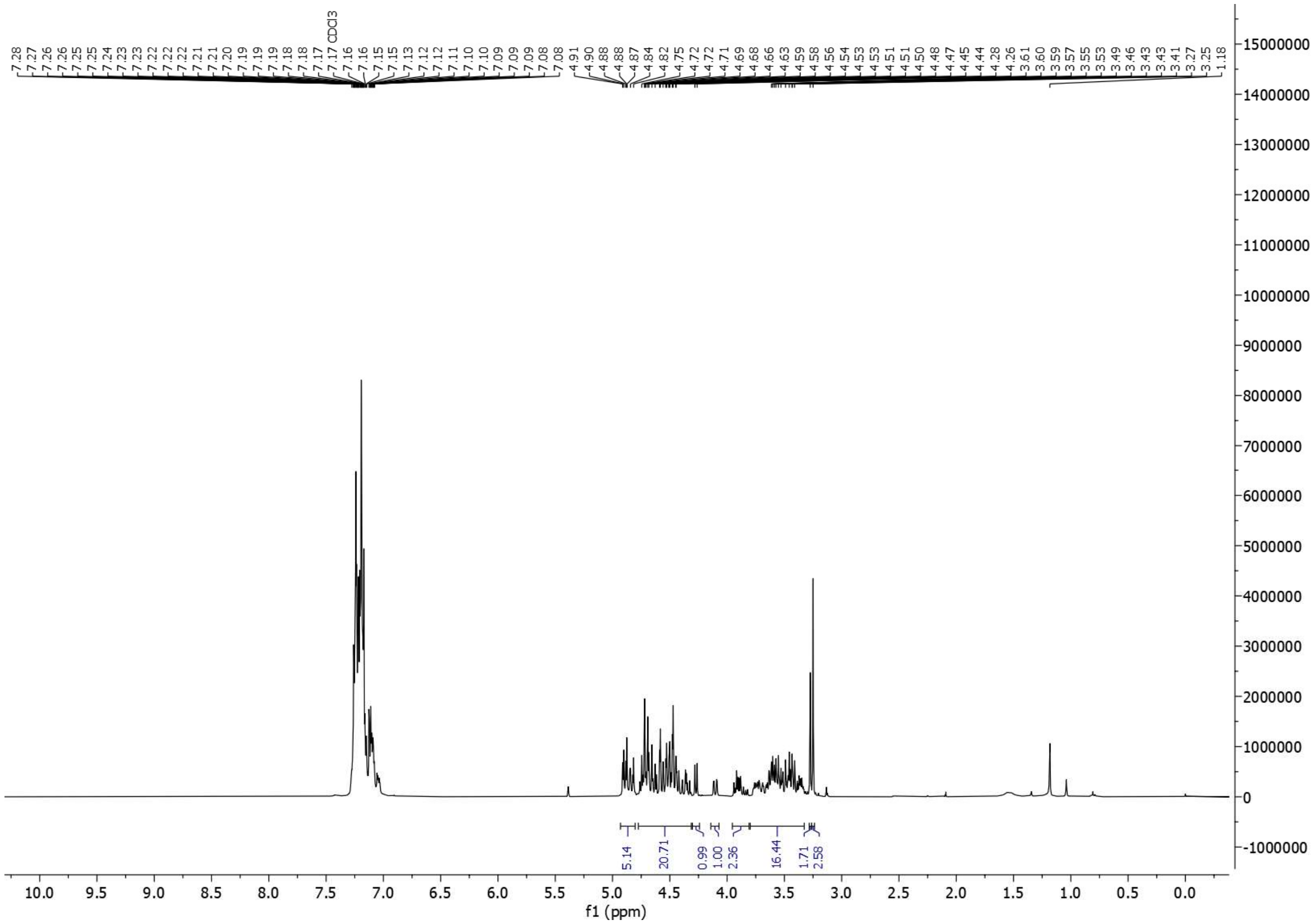

Figure S7. ^{1}H NMR Spectrum ($CDCl_3$, 400 MHz) of Compound **10.**

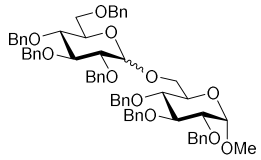

Methyl 2,3,4-tri-*O*-benzyl-6-*O*-(2,3,4,6-tetra-*O*-benzyl-α/β-D-glucopyranosyl)-α-D-glucopyranoside (10)

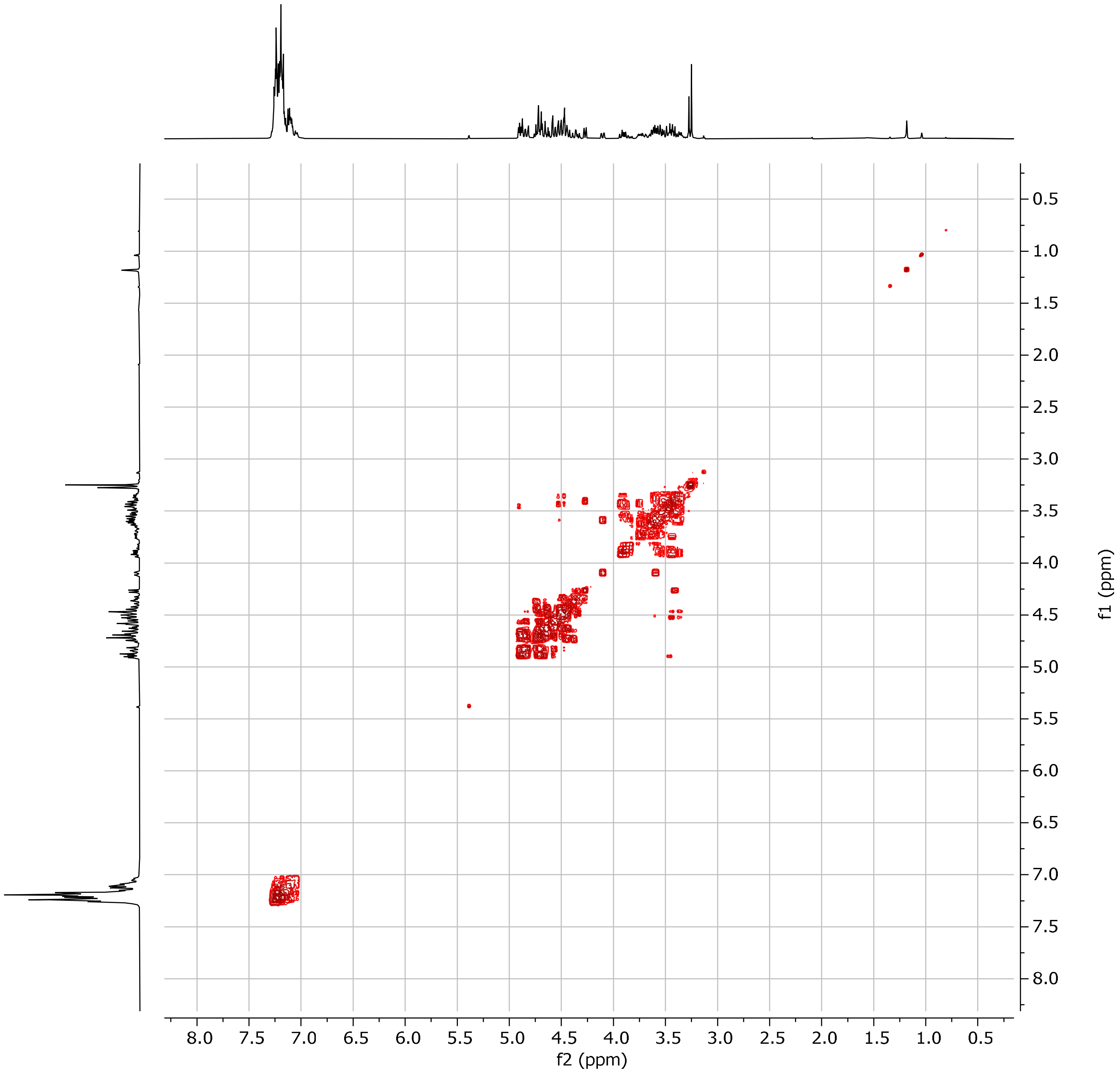

Figure S8. COSY NMR Spectrum ($CDCl_3$, 400 MHz) of Compound **10**.

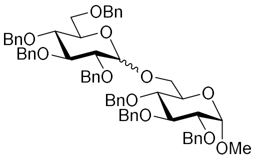

Methyl 2,3,4-tri-*O*-benzyl-6-*O*-(2,3,4,6-tetra-*O*-benzyl-α/β-D-glucopyranosyl)-α-D-glucopyranoside (10)

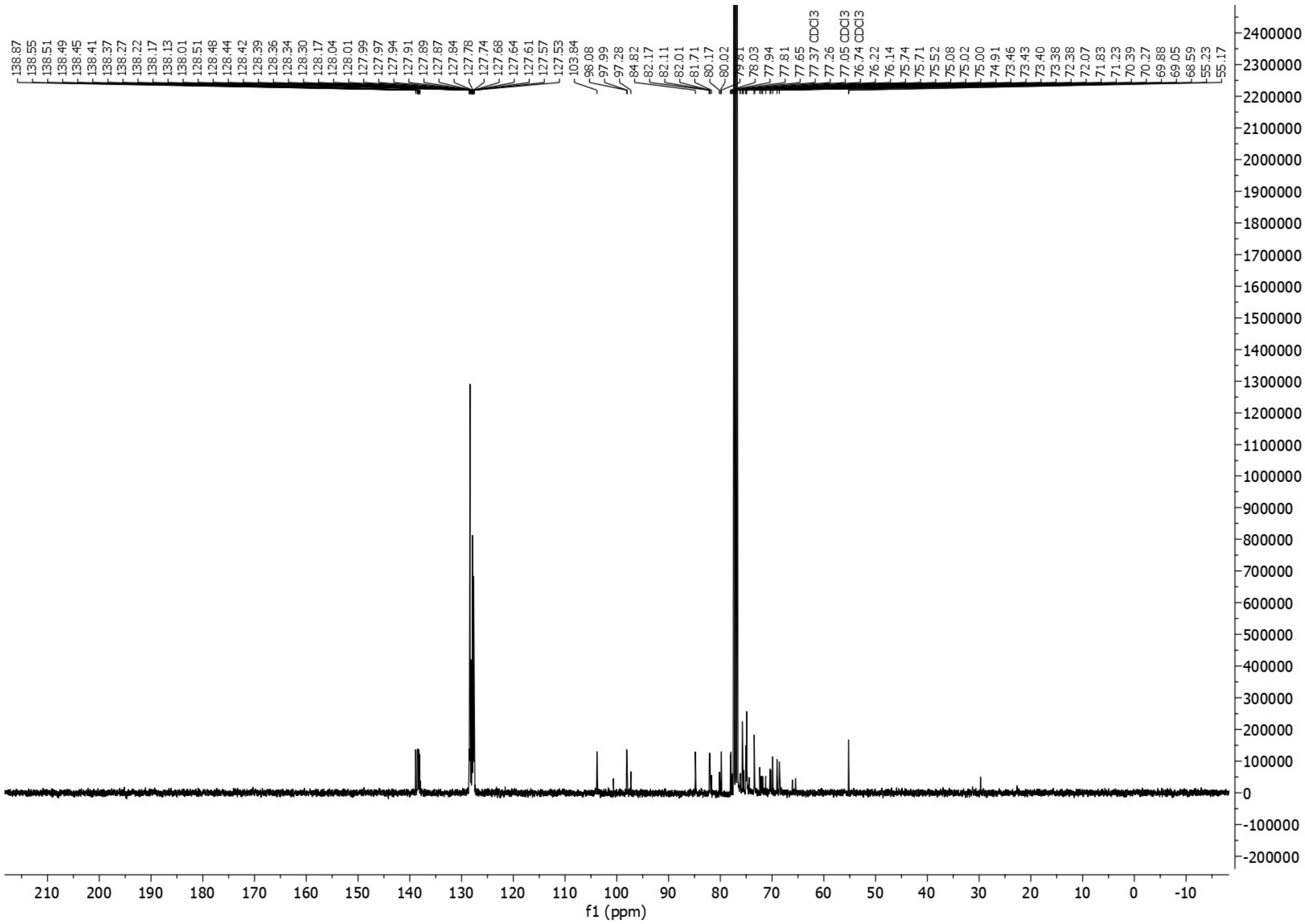

Figure S9. ^{13}C NMR Spectrum ($CDCl_3$, 101 MHz) of Compound **10**.

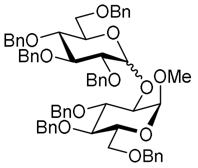

Methyl 3,4,6-tri-*O*-benzyl-2-*O*-(2,3,4,6-tetra-*O*-benzyl-α/β-D-glucopyranosyl)-α-D-glucopyranoside (11)

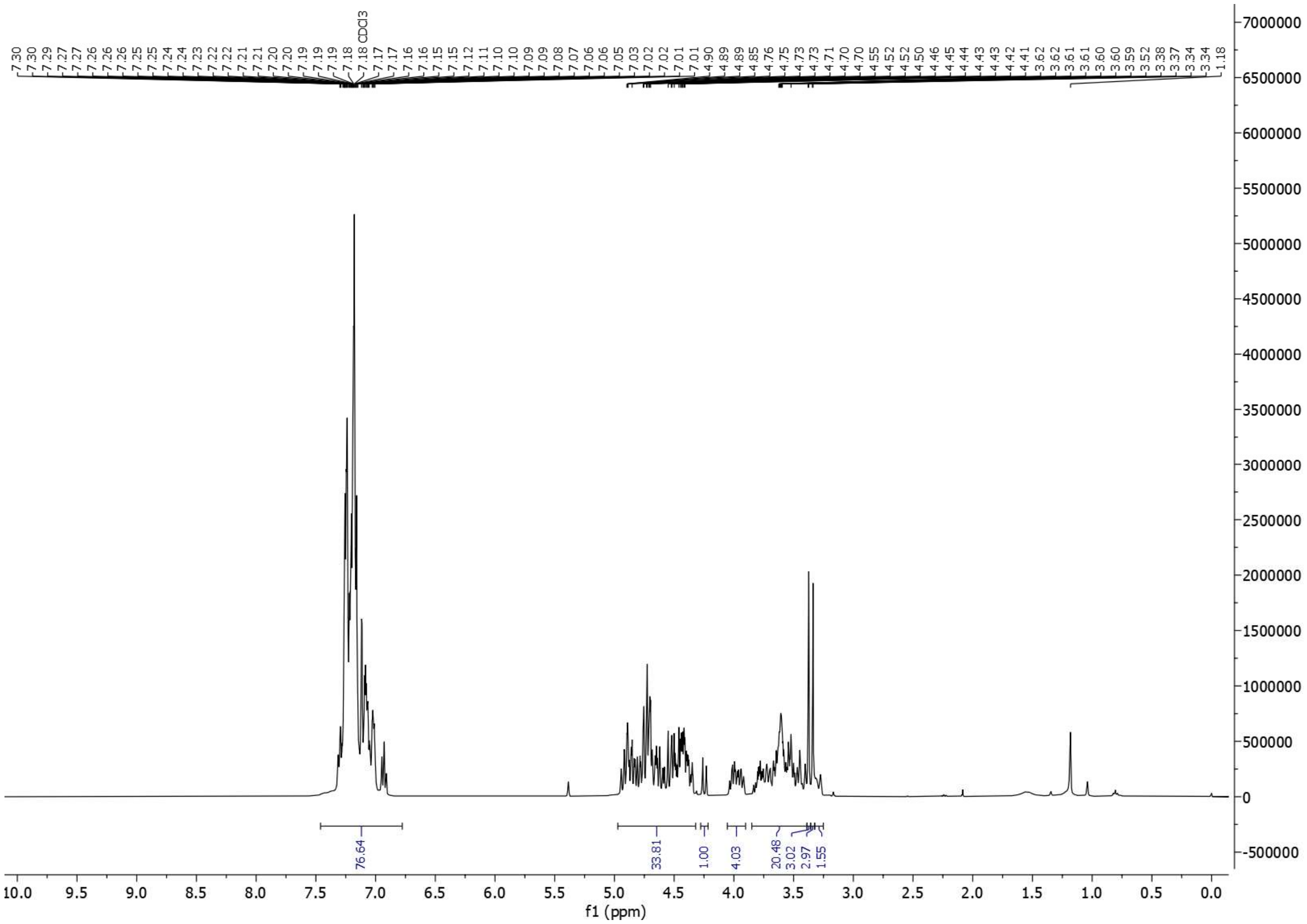

Figure S10. ^{1}H NMR Spectrum ($CDCl_3$, 400 MHz) of Compound **11.**

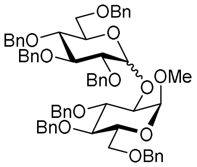

Methyl 3,4,6-tri-*O*-benzyl-2-*O*-(2,3,4,6-tetra-*O*-benzyl-α/β-D-glucopyranosyl)-α-D-glucopyranoside (11)

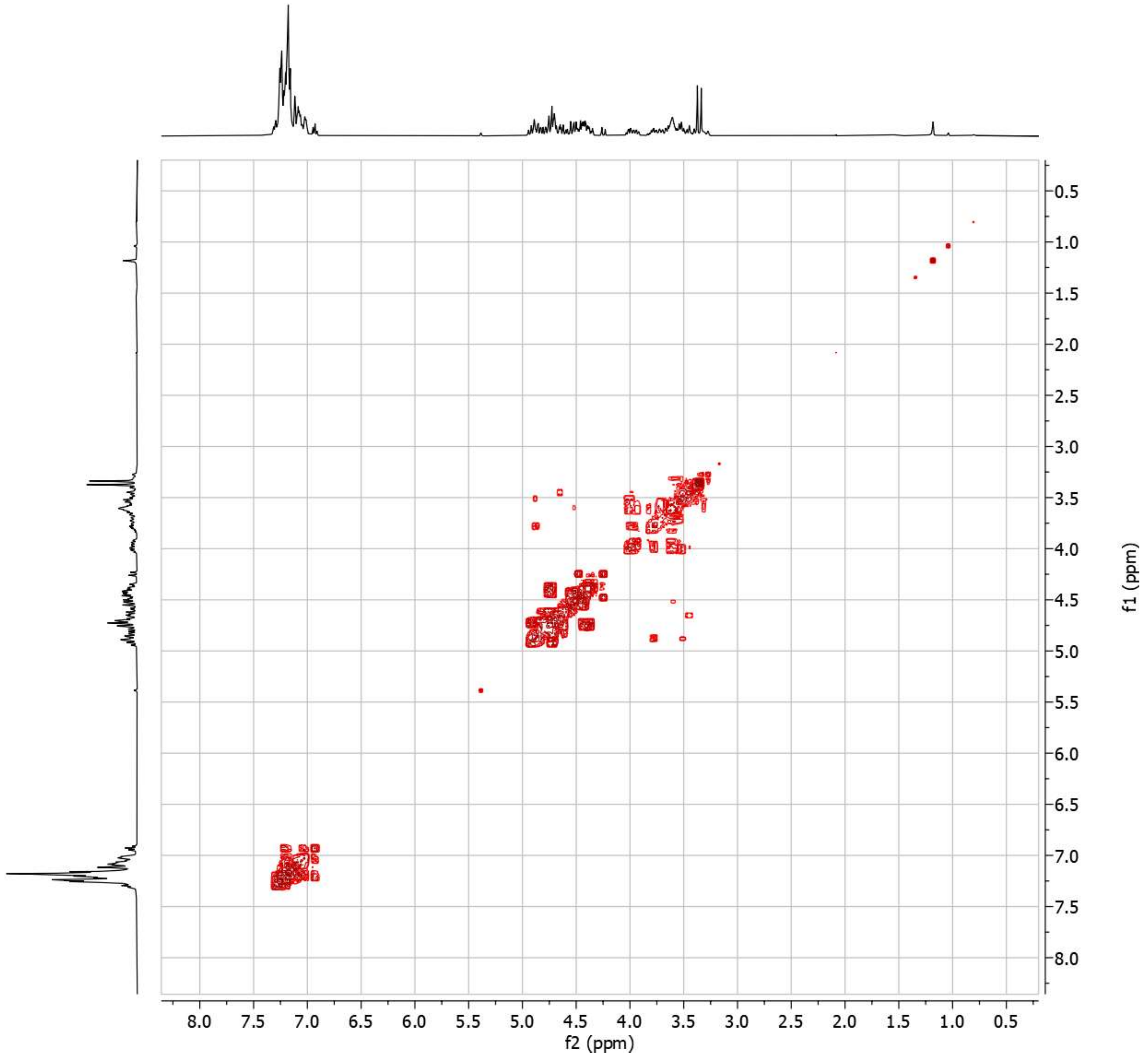

Figure S11. COSY NMR Spectrum ($CDCl_3$, 400 MHz) of Compound **11**.

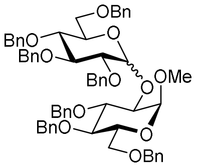

Methyl 3,4,6-tri-*O*-benzyl-2-*O*-(2,3,4,6-tetra-*O*-benzyl-α/β-D-glucopyranosyl)-α-D-glucopyranoside (11)

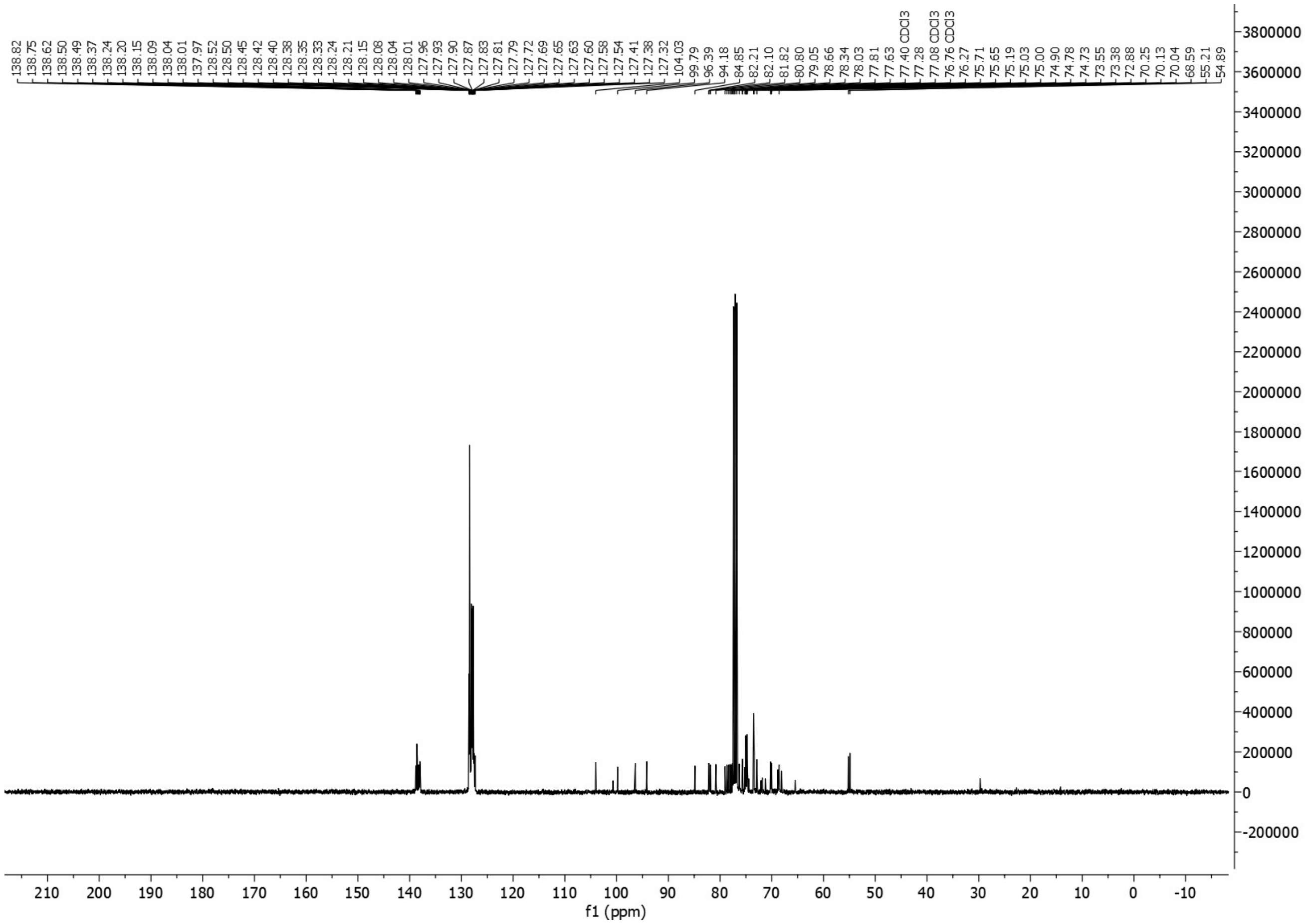

Figure S12. ^{13}C NMR Spectrum ($CDCl_3$, 101 MHz) of Compound **11**.

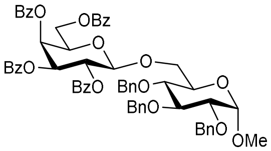

Methyl 6-*O*-(2,3,4,6-tetra-*O*-benzoyl-β-D-galactopyranosyl)-2,3,4-tri-*O*-benzyl-α-D-glucopyranoside (13)

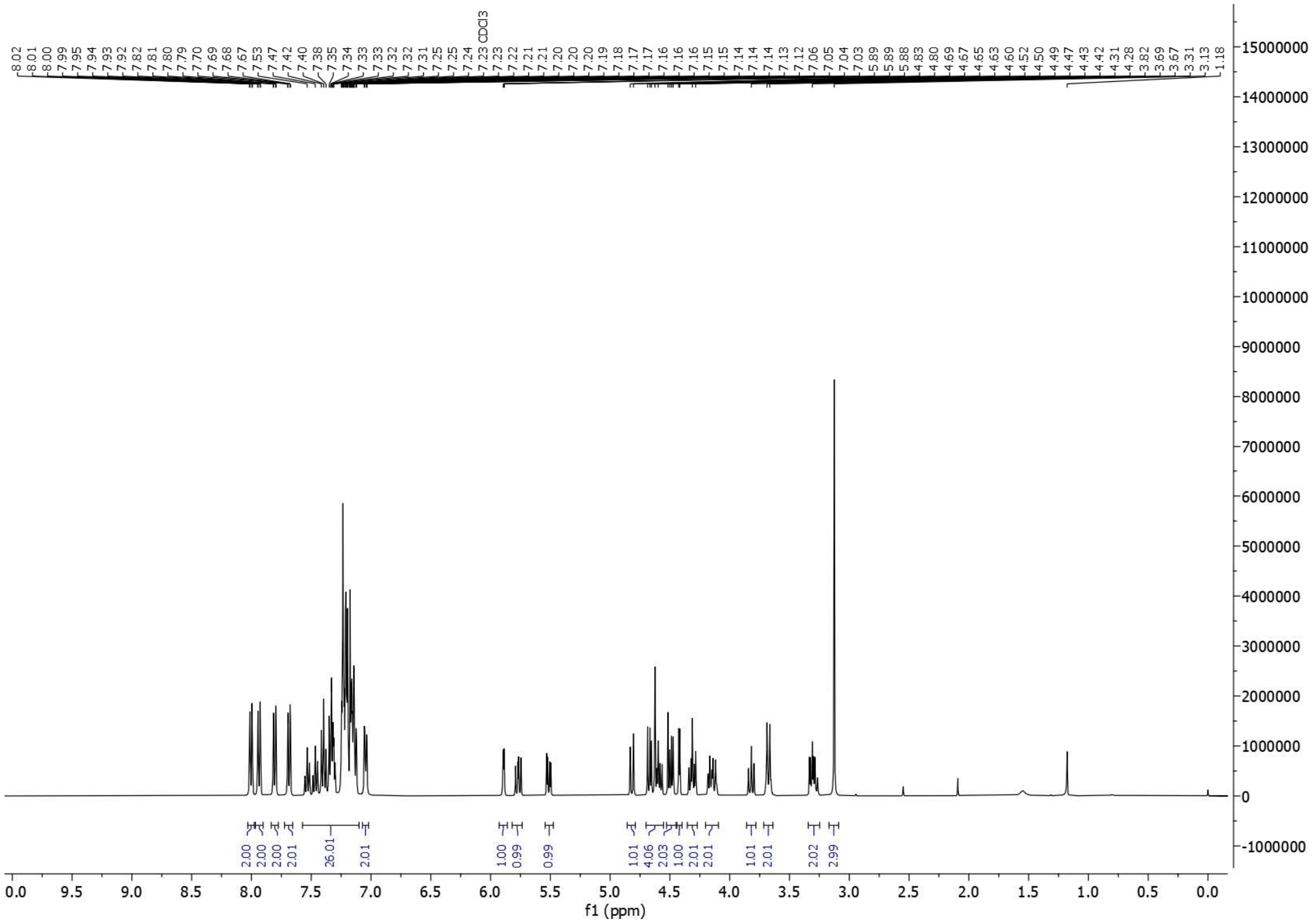

Figure S13. ^{1}H NMR Spectrum ($CDCl_3$, 400 MHz) of Compound **13.**

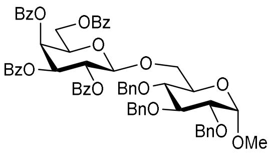

Methyl 6-*O*-(2,3,4,6-tetra-*O*-benzoyl-β-D-galactopyranosyl)-2,3,4-tri-*O*-benzyl-α-D-glucopyranoside (13)

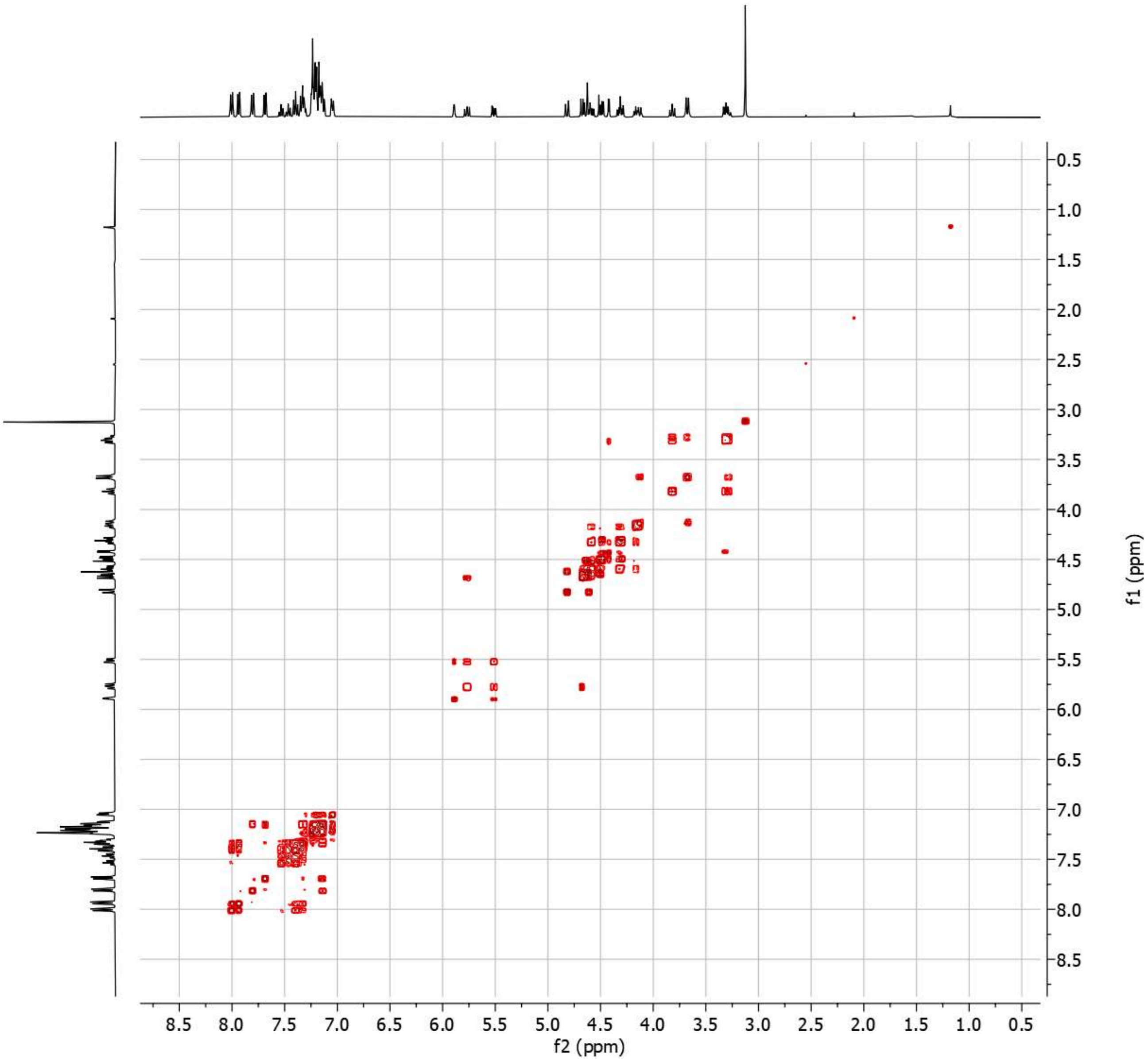

Figure S14. COSY NMR Spectrum ($CDCl_3$, 400 MHz) of Compound **13.**

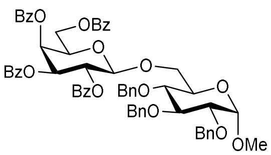

Methyl 6-*O*-(2,3,4,6-tetra-*O*-benzoyl-β-D-galactopyranosyl)-2,3,4-tri-*O*-benzyl-α-D-glucopyranoside (13)

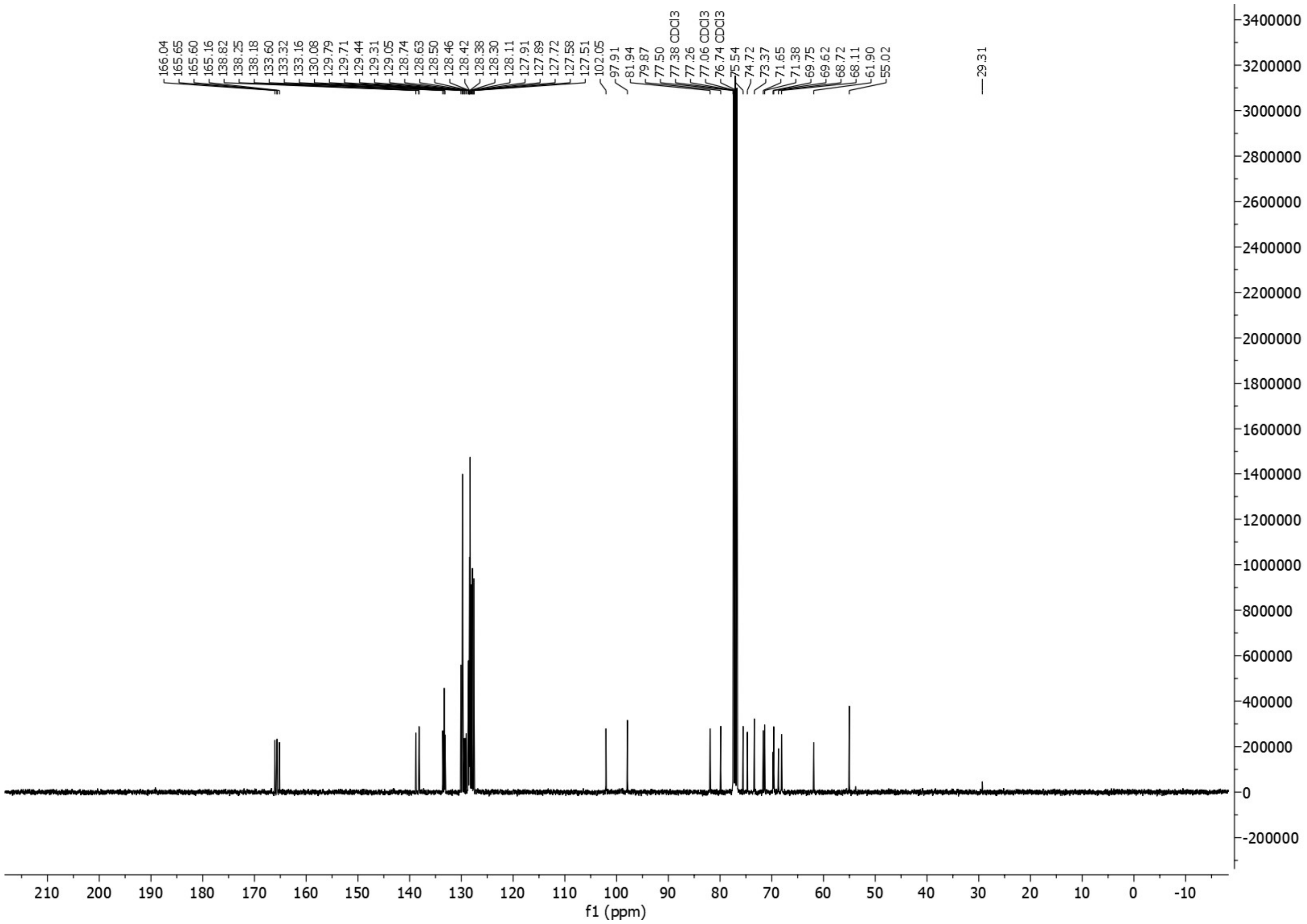

Figure S15. ^{13}C NMR Spectrum ($CDCl_3$, 101 MHz) of Compound **13.**

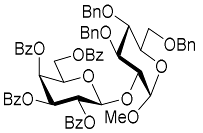

Methyl 2-*O*-(2,3,4,6-tetra-*O*-benzoyl-β-D-galactopyranosyl)-3,4,6-tri-*O*-benzyl-*α*-D-glucopyranoside (14)

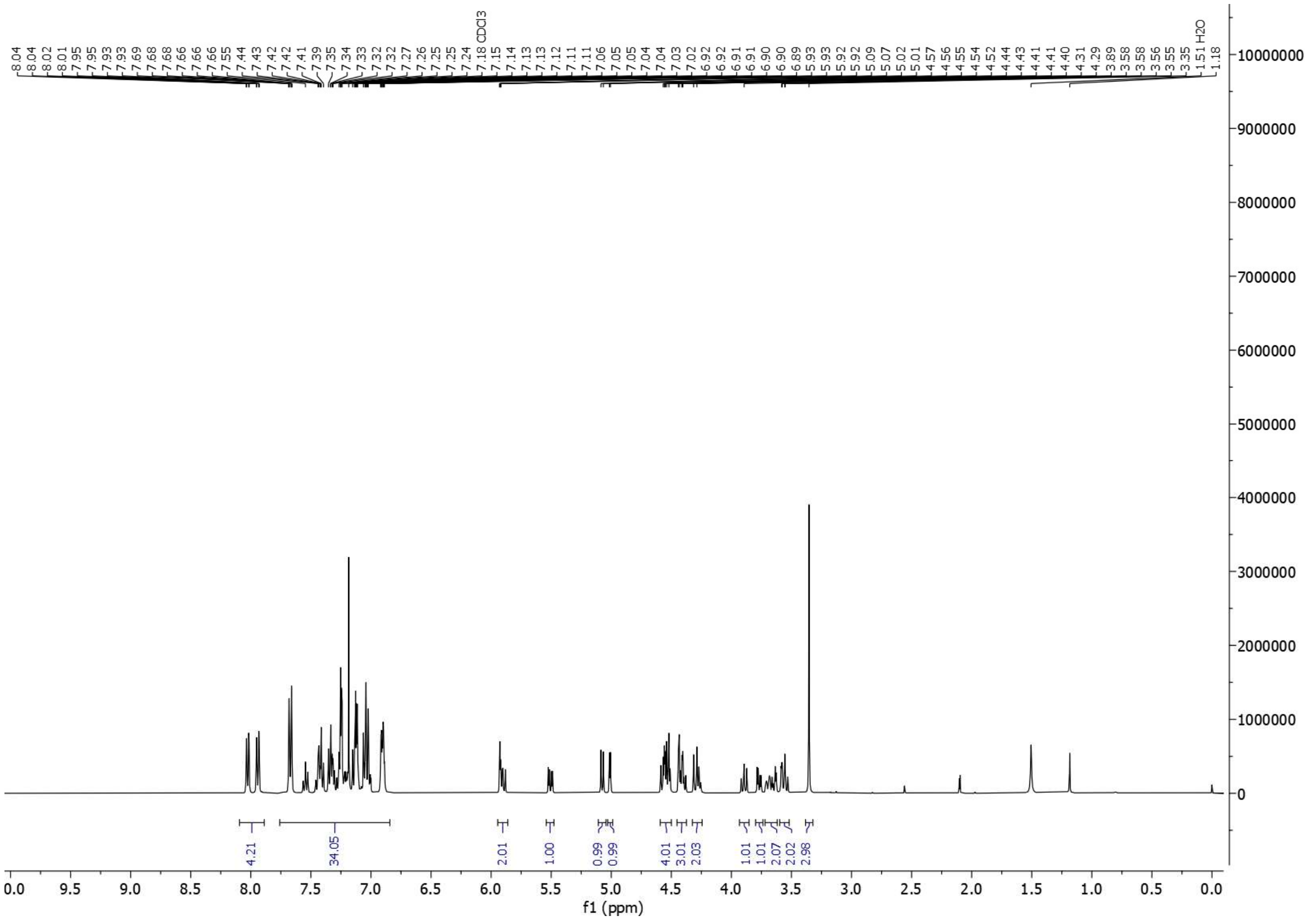

Figure S16. ^{1}H NMR Spectrum ($CDCl_3$, 400 MHz) of Compound **14.**

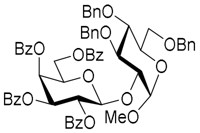

Methyl 2-*O*-(2,3,4,6-tetra-*O*-benzoyl-β-D-galactopyranosyl)-3,4,6-tri-*O*-benzyl-α-D-glucopyranoside (14)

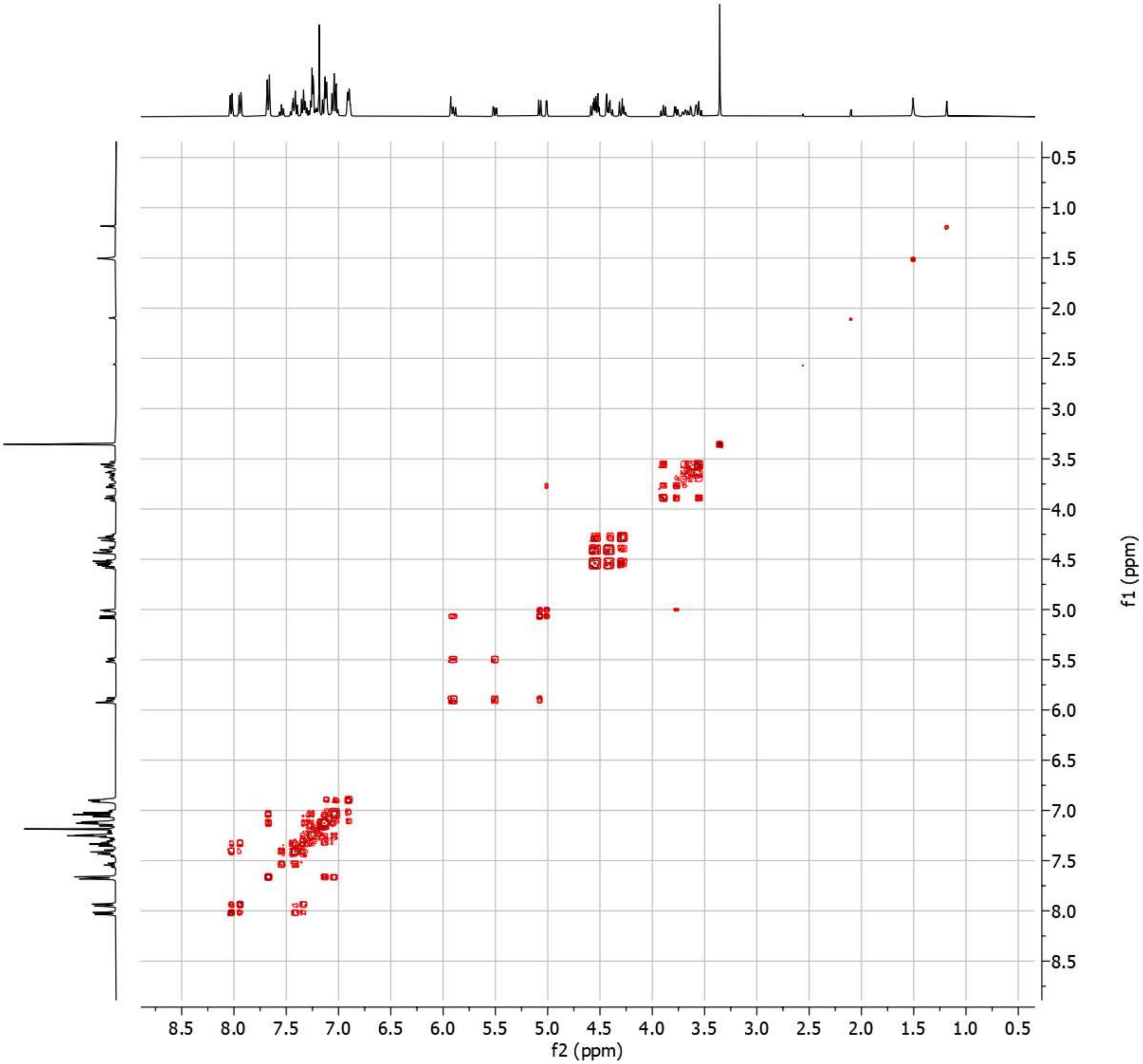

Figure S17. COSY NMR Spectrum ($CDCl_3$, 400 MHz) of Compound **14**.

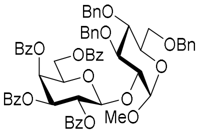

Methyl 2-*O*-(2,3,4,6-tetra-*O*-benzoyl-β-D-galactopyranosyl)-3,4,6-tri-*O*-benzyl-α-D-glucopyranoside (14)

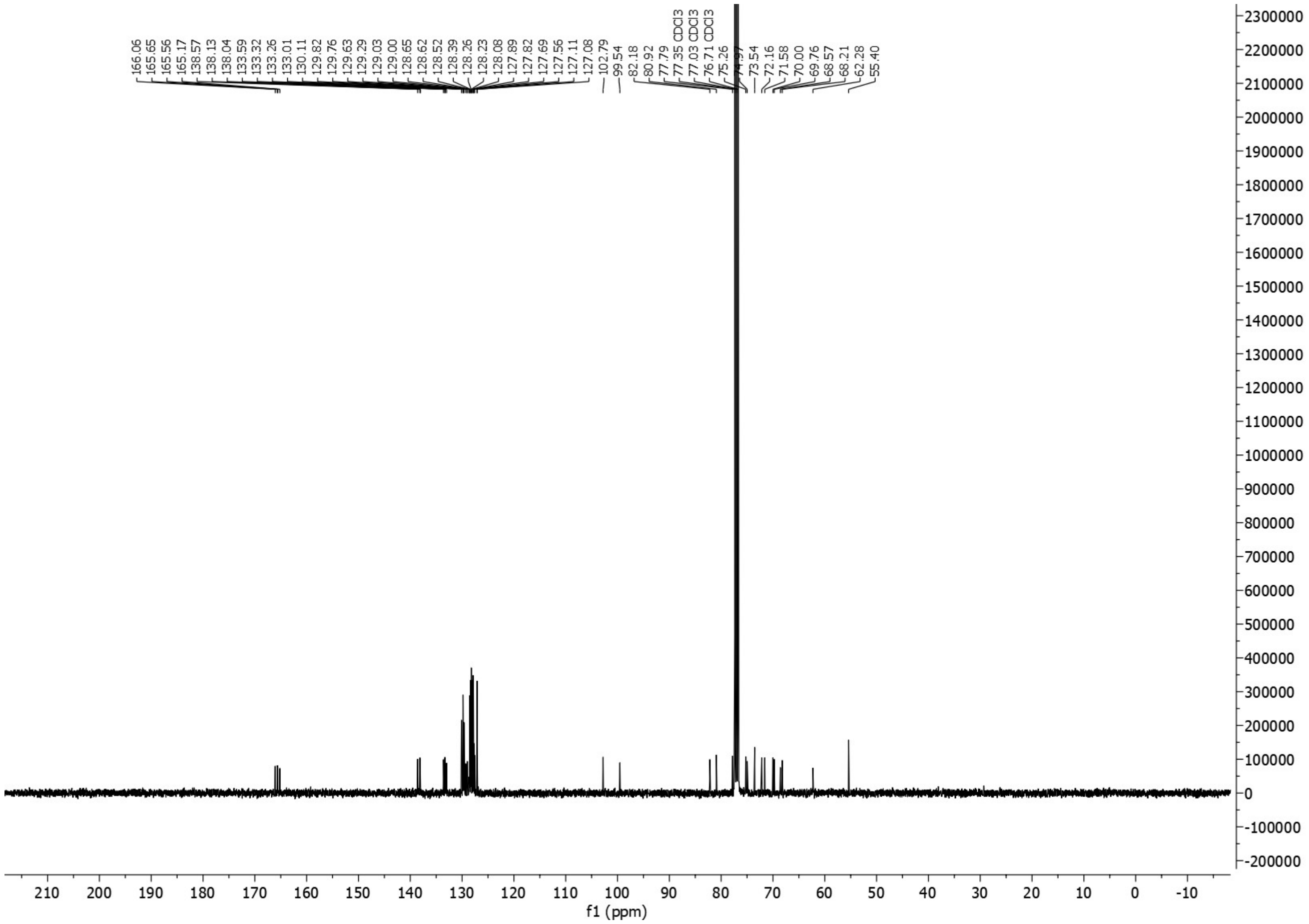

Figure S18. ^{13}C NMR Spectrum ($CDCl_3$, 101 MHz) of Compound **14**.

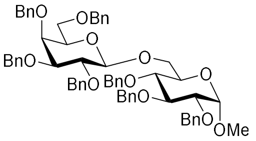

Methyl 2,3,4-tri-*O*-benzyl-6-*O*-(2,3,4,6-tetra-*O*-benzyl-β-D-galactopyranosyl)-α-D-glucopyranoside (16)

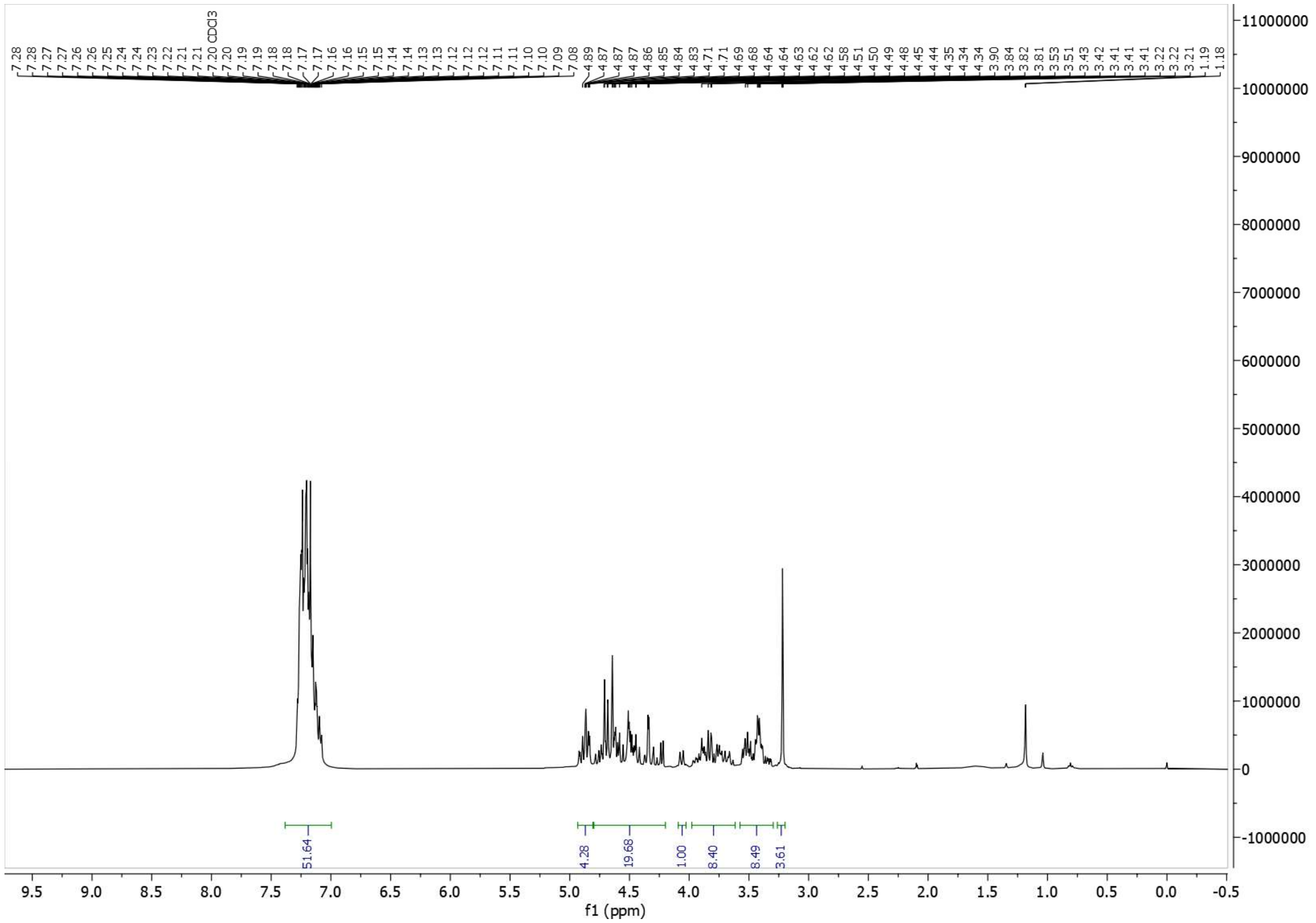

Figure S19. ^{1}H NMR Spectrum ($CDCl_3$, 400 MHz) of Compound **16.**

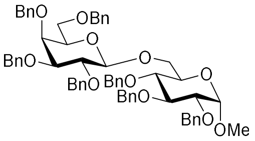

Methyl 2,3,4-tri-*O*-benzyl-6-*O*-(2,3,4,6-tetra-*O*-benzyl-β-D-galactopyranosyl)-*α*-D-glucopyranoside (16)

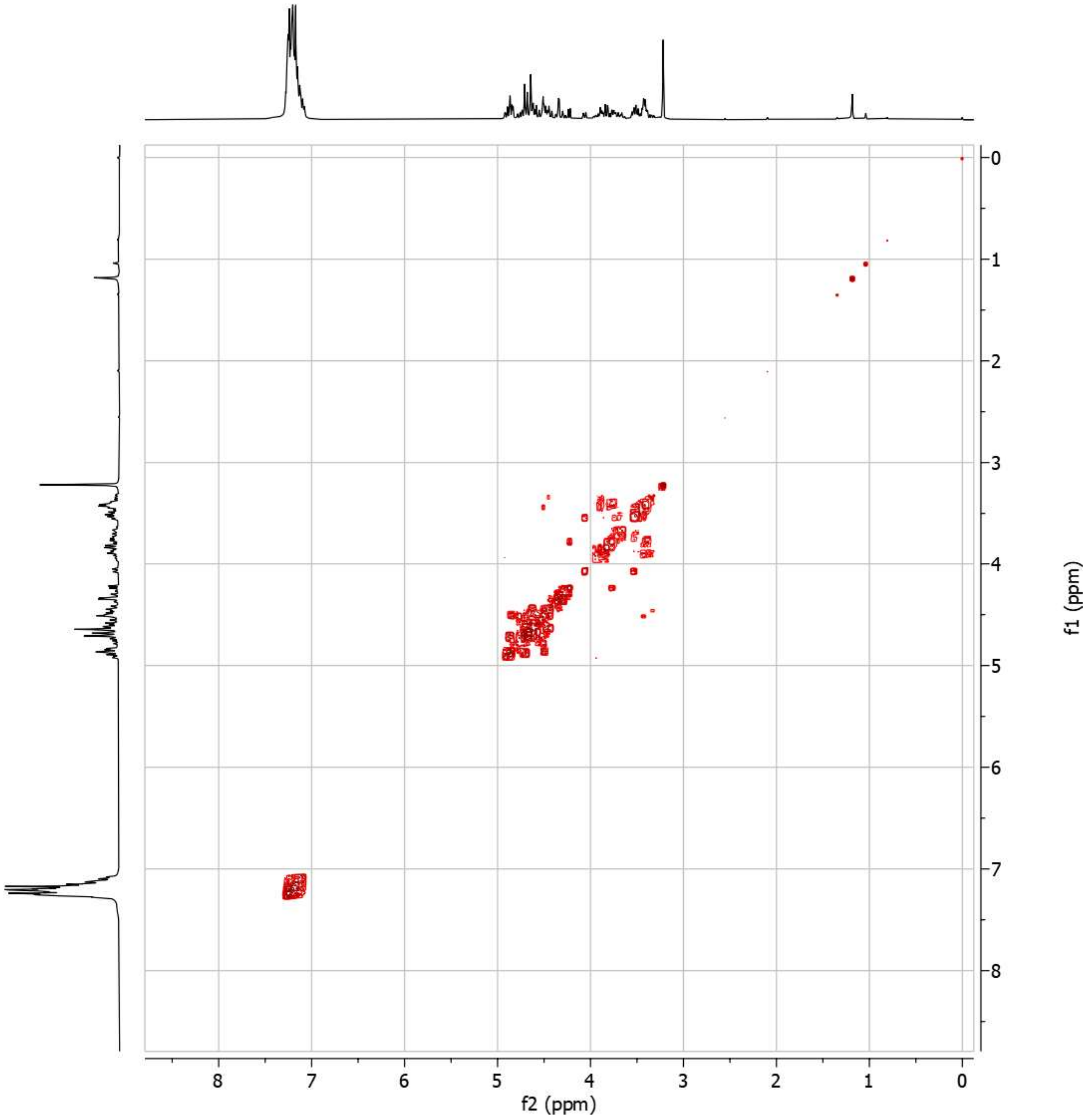

Figure S20. COSY NMR Spectrum ($CDCl_3$, 400 MHz) of Compound **16**.

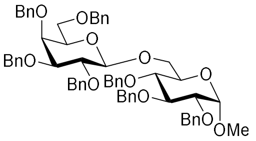

Methyl 2,3,4-tri-*O*-benzyl-6-*O*-(2,3,4,6-tetra-*O*-benzyl-β-D-galactopyranosyl)-*α*-D-glucopyranoside (16)

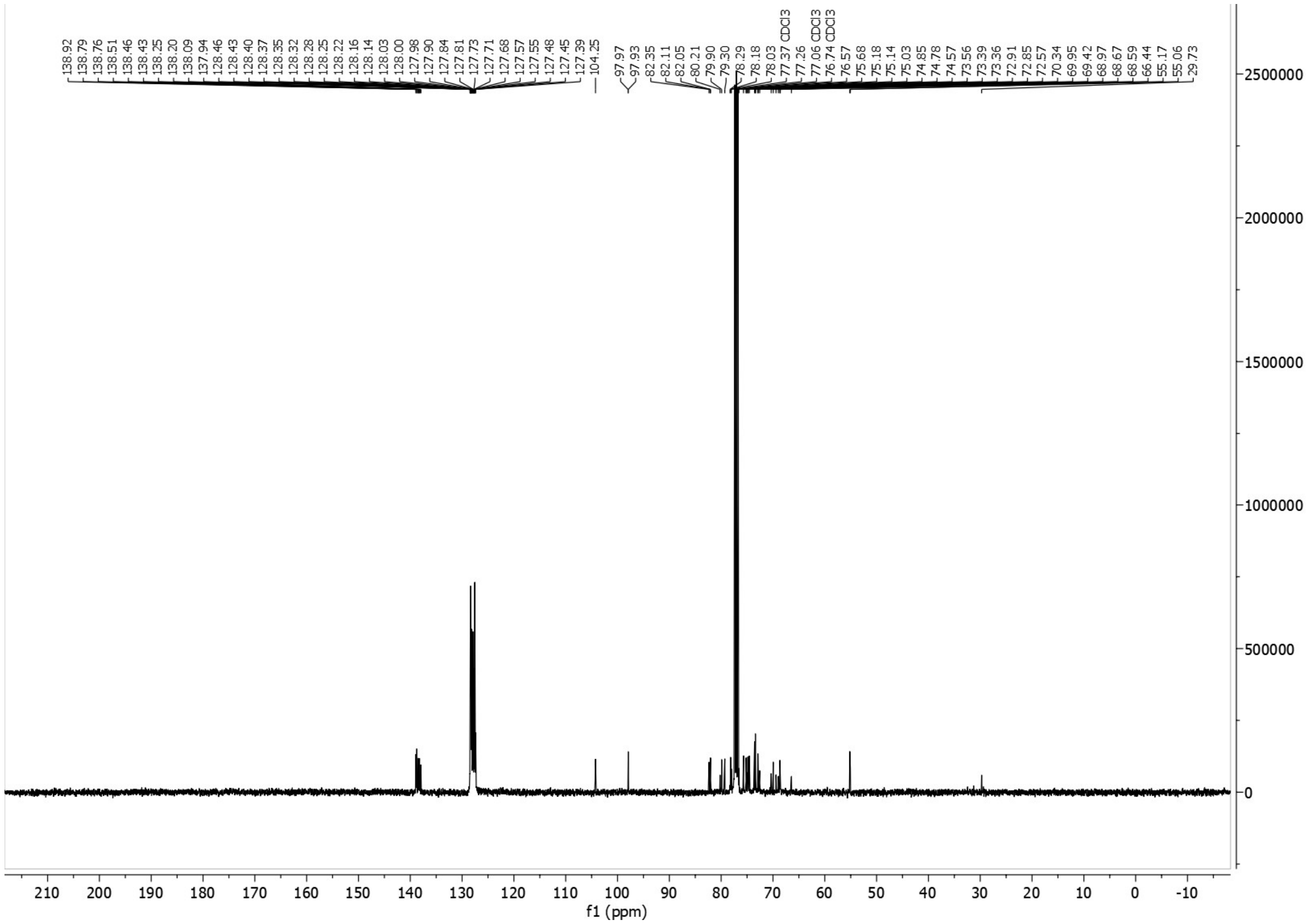

Figure S21. ^{13}C NMR Spectrum ($CDCl_3$, 101 MHz) of Compound **16**.

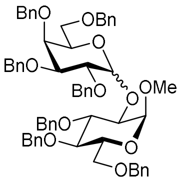

Methyl 3,4,6-tri-*O*-benzyl-2-*O*-(2,3,4,6-tetra-*O*-benzyl-α/β-D-galactopyranosyl)-α-D-glucopyranoside (17)

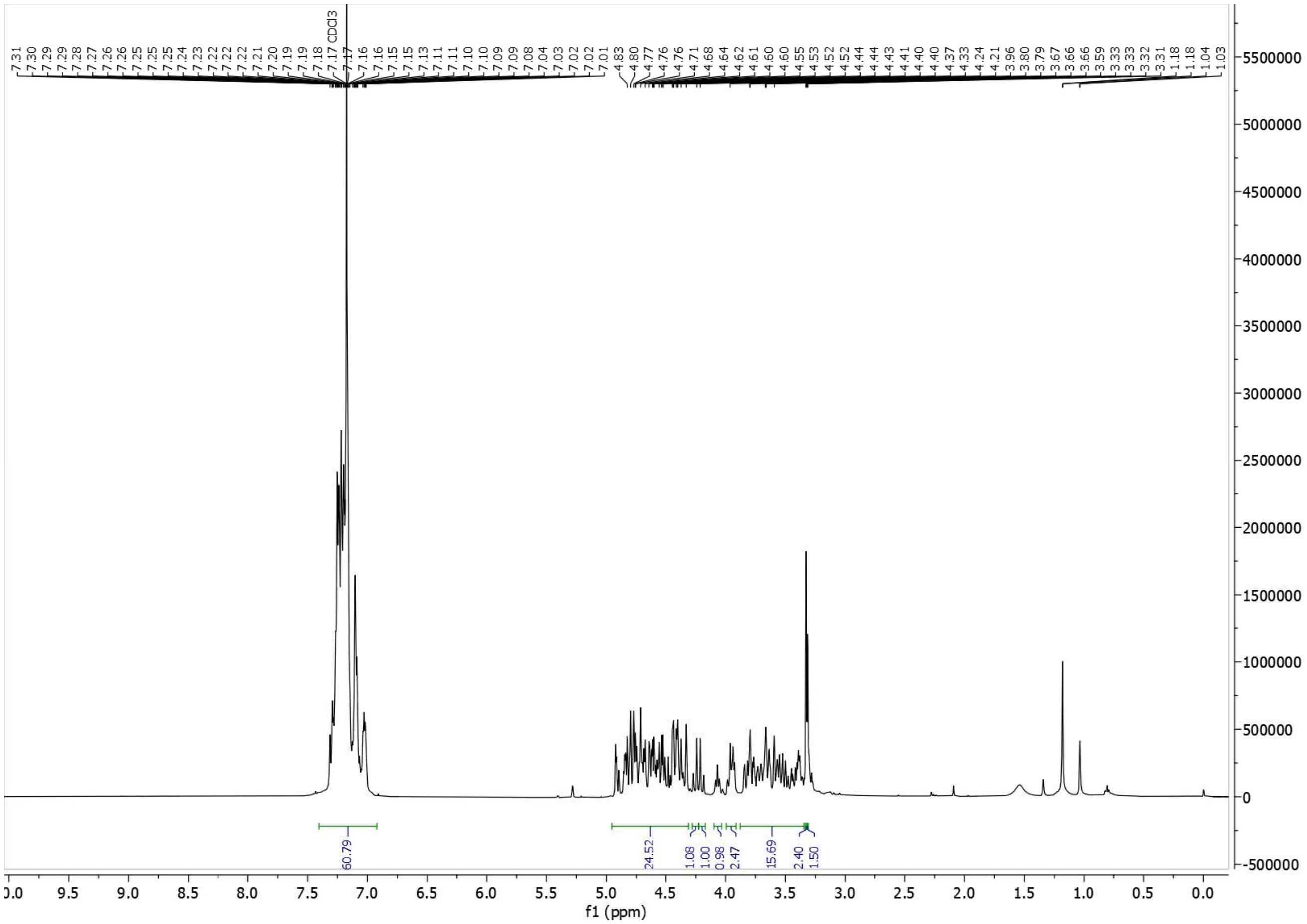

Figure S22. ^{1}H NMR Spectrum ($CDCl_3$, 400 MHz) of Compound **17.**

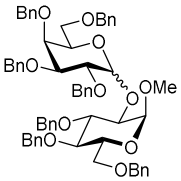

Methyl 3,4,6-tri-*O*-benzyl-2-*O*-(2,3,4,6-tetra-*O*-benzyl-α/β-D-galactopyranosyl)-α-D-glucopyranoside (17)

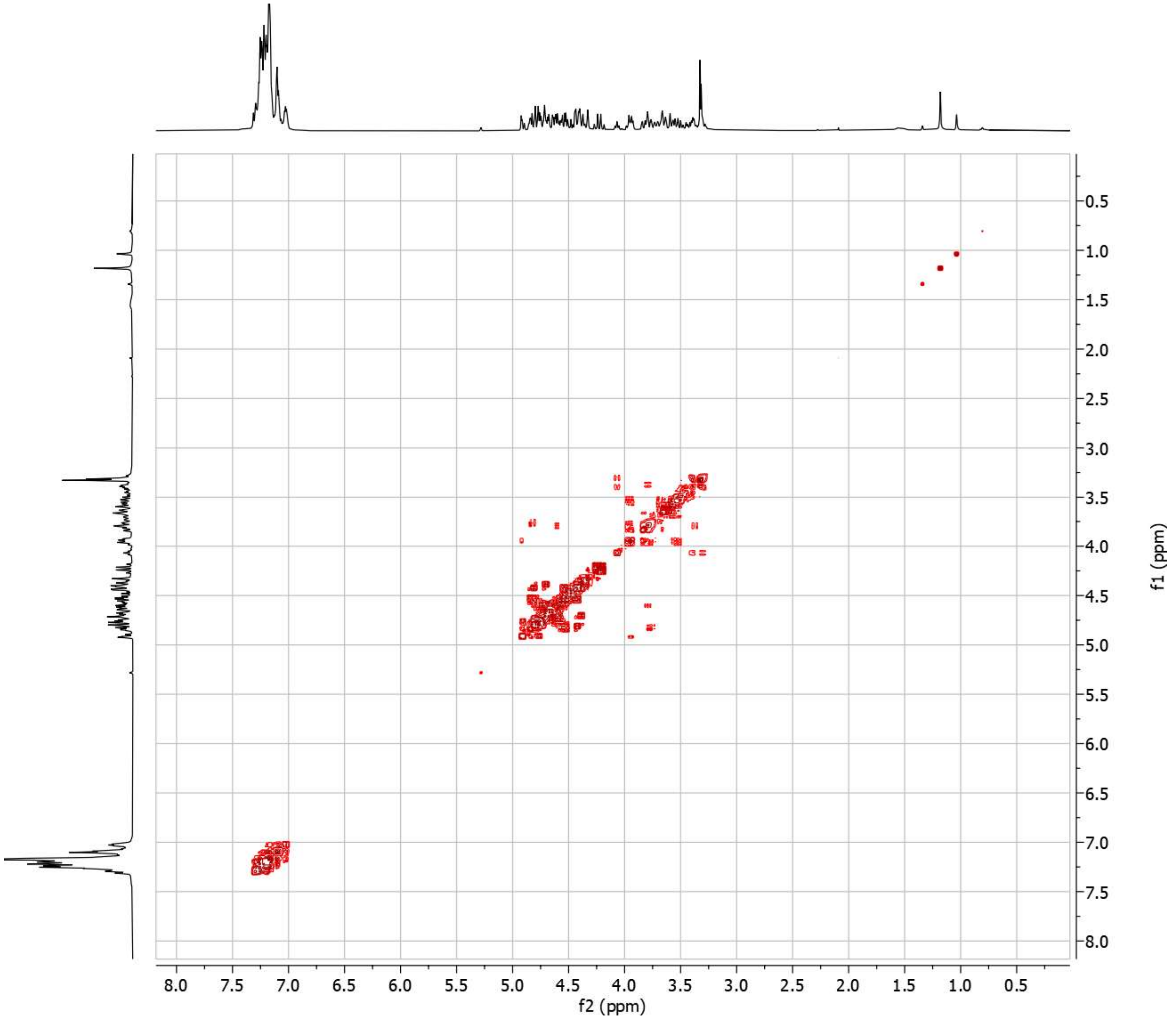

Figure S23. COSY NMR Spectrum ($CDCl_3$, 400 MHz) of Compound **17**.

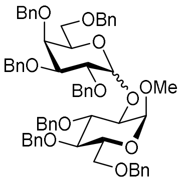

Methyl 3,4,6-tri-*O*-benzyl-2-*O*-(2,3,4,6-tetra-*O*-benzyl-*α*/β-D-galactopyranosyl)-*α*-D-glucopyranoside (17)

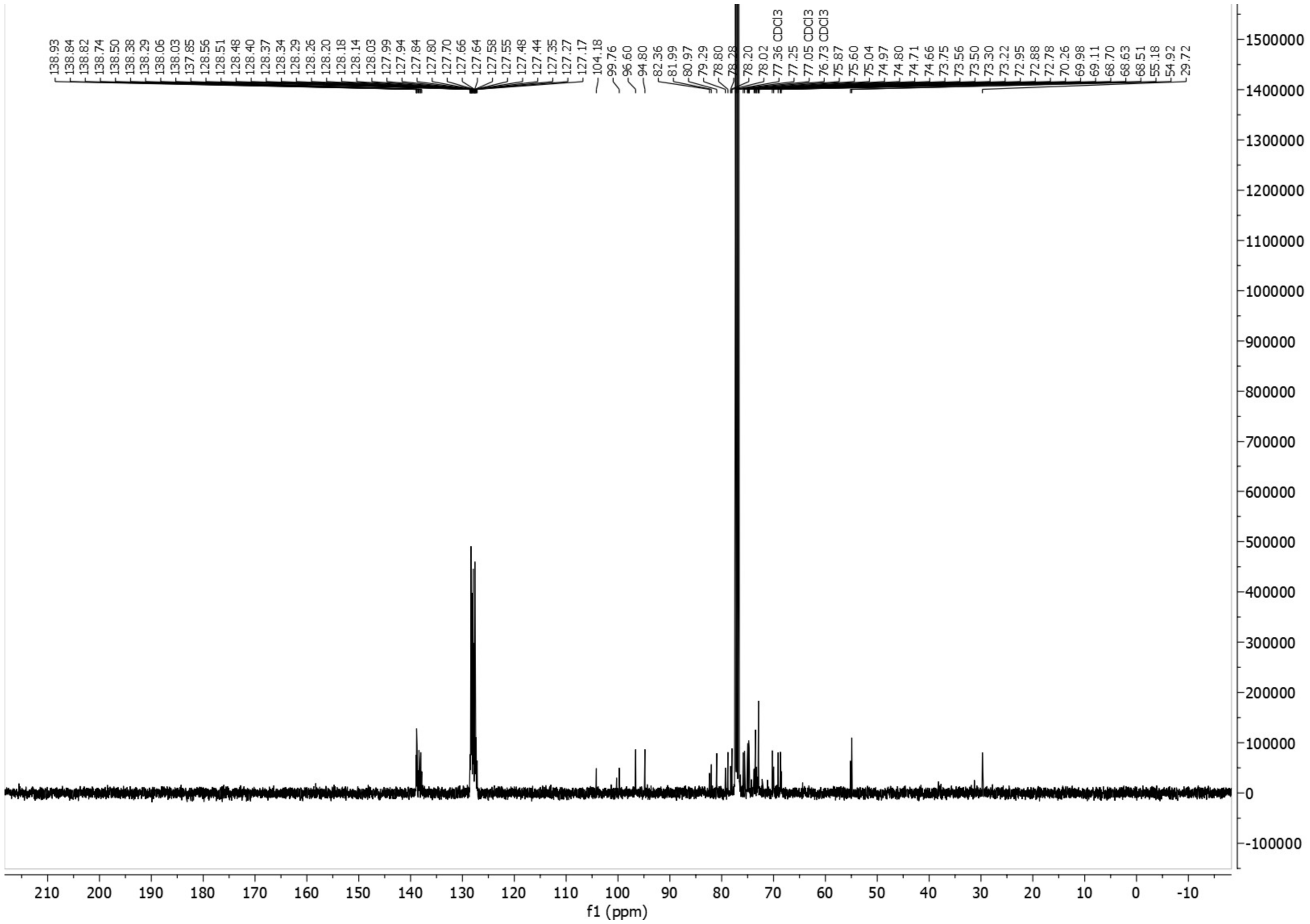

Figure S24. ^{13}C NMR Spectrum ($CDCl_3$, 101 MHz) of Compound **17**.

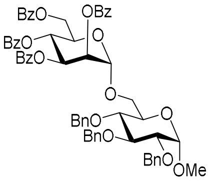

Methyl 6-*O*-(2,3,4,6-tetra-*O*-benzoyl-*α*-D-mannopyranosyl)-2,3,4-tri-*O*-benzyl-*α*-D-glucopyranoside (19)

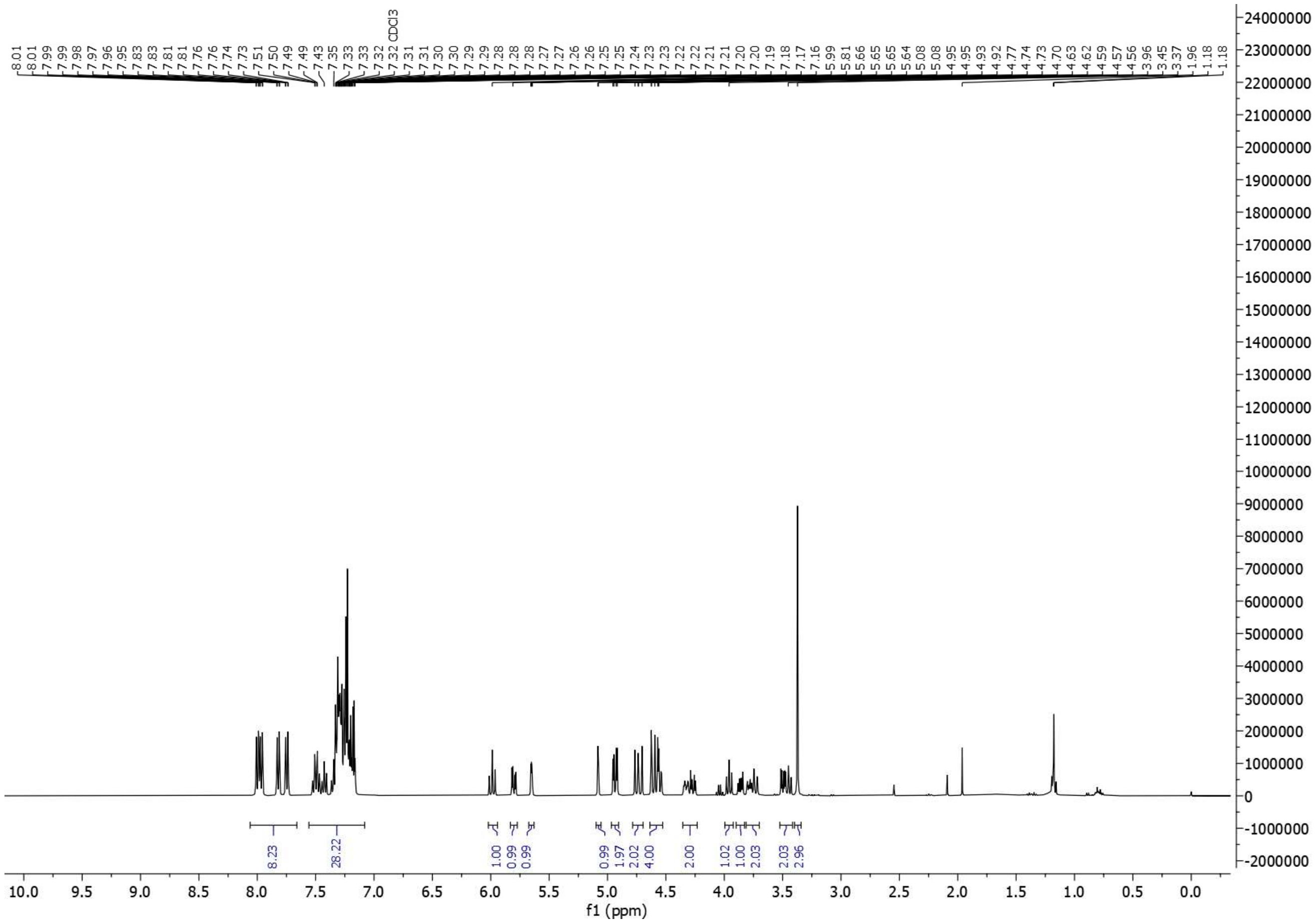

Figure S25. ^{1}H NMR Spectrum ($CDCl_3$, 400 MHz) of Compound **19.**

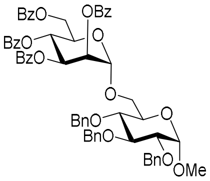

Methyl 6-*O*-(2,3,4,6-tetra-*O*-benzoyl-*α*-D-mannopyranosyl)-2,3,4-tri-*O*-benzyl-*α*-D-glucopyranoside (19)

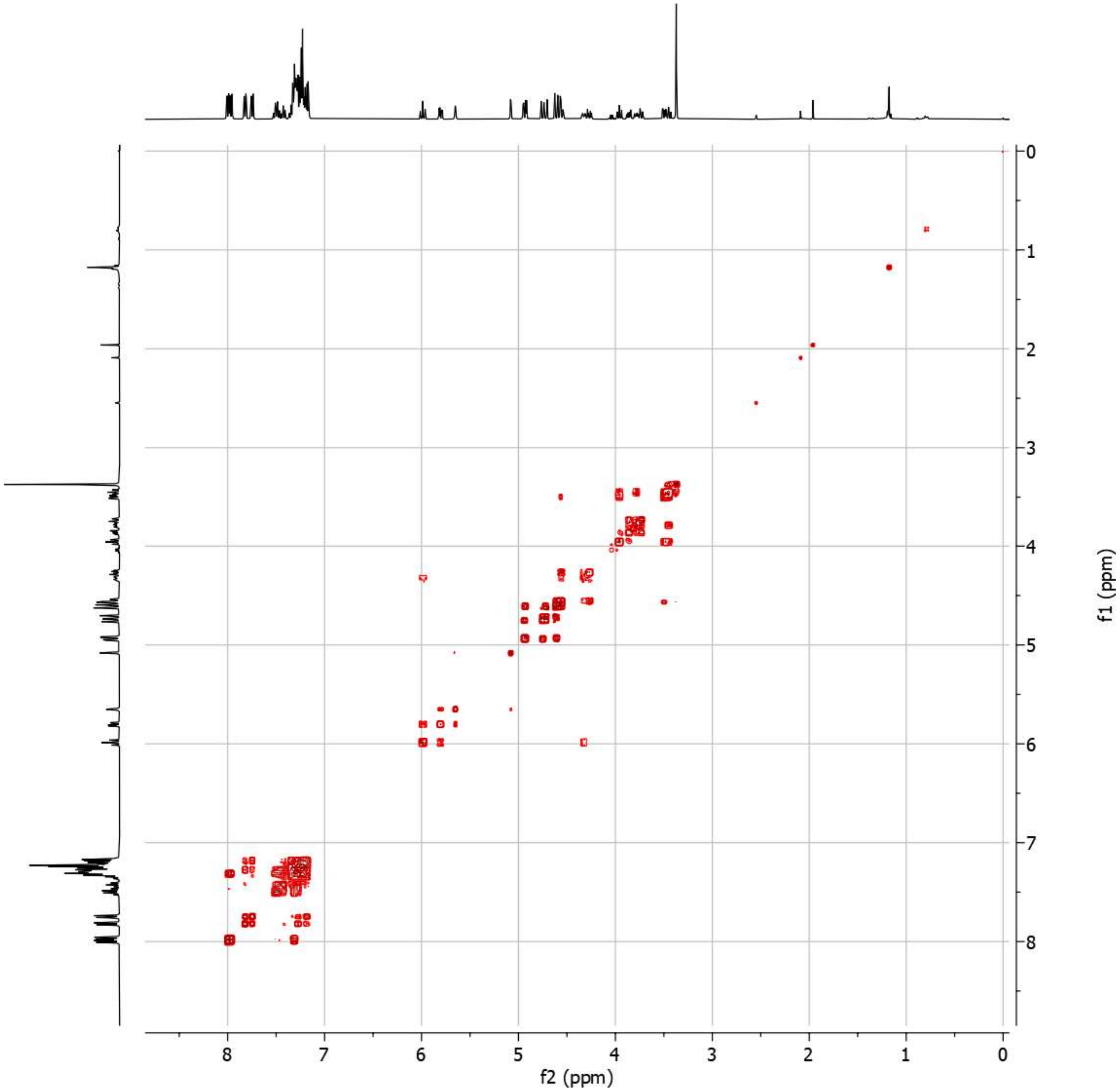

Figure S26. COSY NMR Spectrum ($CDCl_3$, 400 MHz) of Compound **19**.

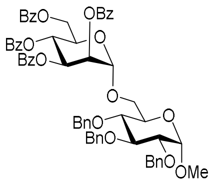

Methyl 6-*O*-(2,3,4,6-tetra-*O*-benzoyl-α-D-mannopyranosyl)-2,3,4-tri-*O*-benzyl-α-D-glucopyranoside (19)

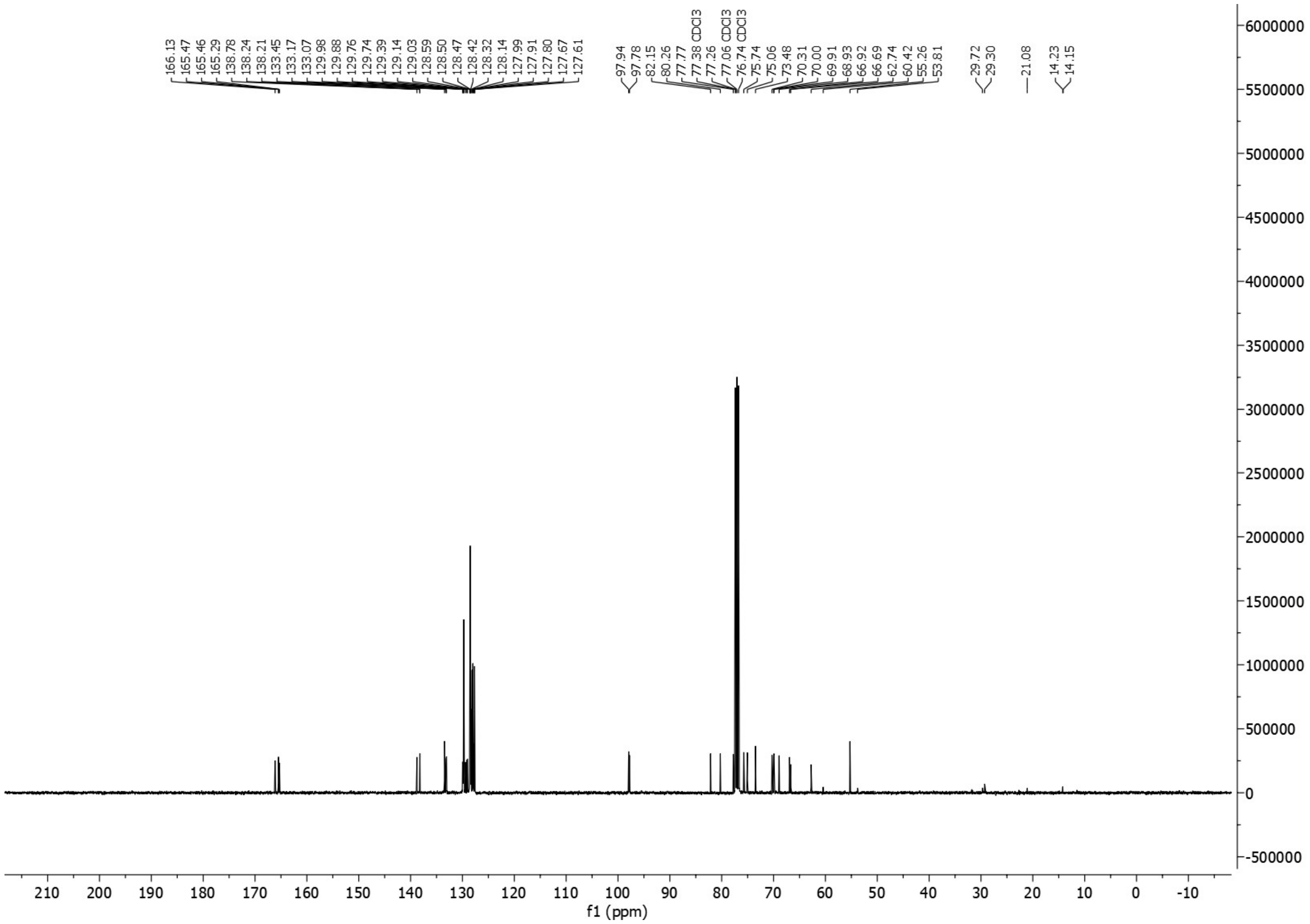

Figure S27. ^{13}C NMR Spectrum ($CDCl_3$, 101 MHz) of Compound **19**.

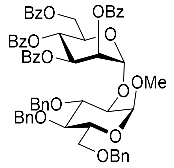

Methyl 2-*O*-(2,3,4,6-tetra-*O*-benzoyl-α-D-mannopyranosyl)-3,4,6-tri-*O*-benzyl-α-D-glucopyranoside (20)

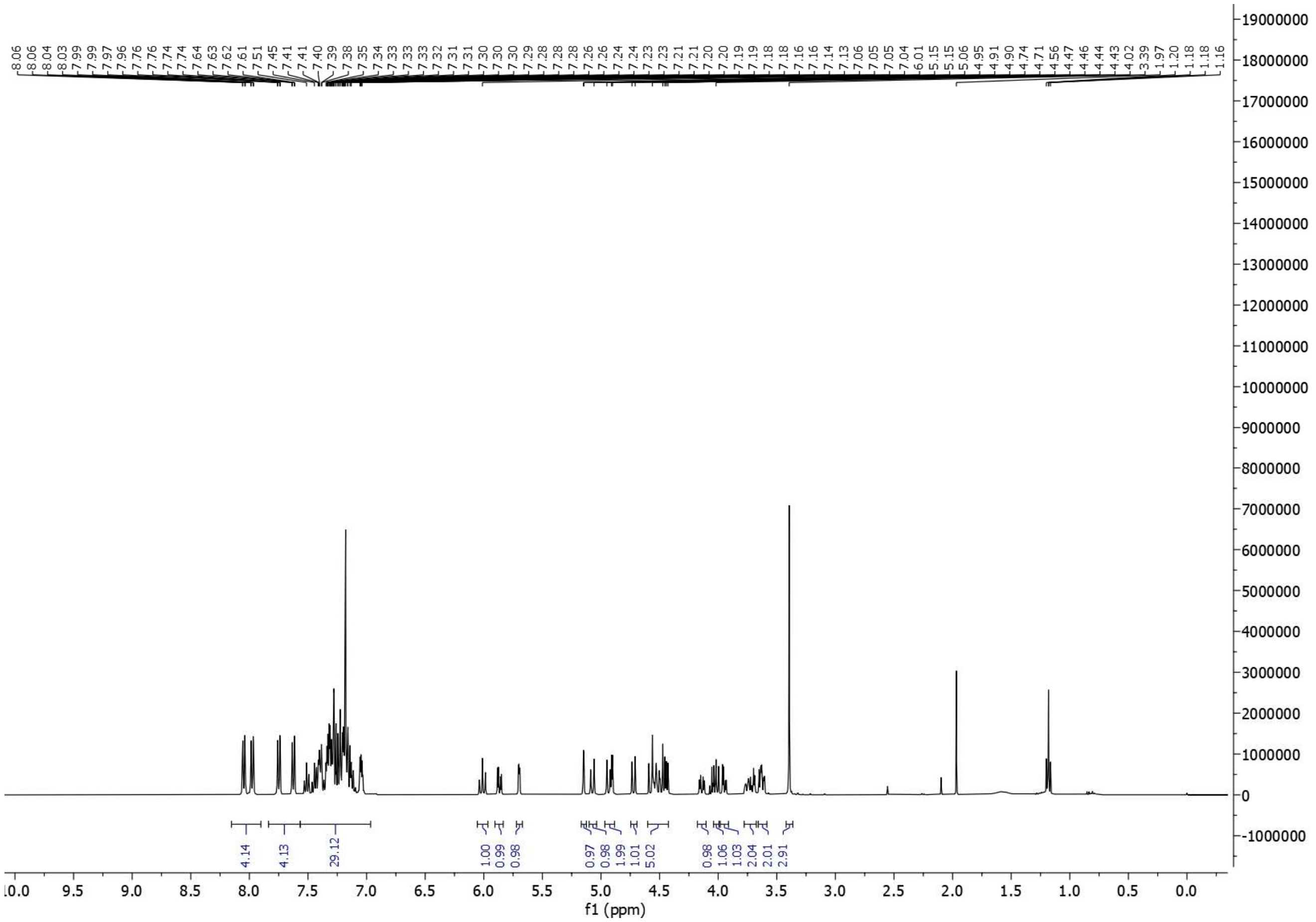

Figure S28. ^{1}H NMR Spectrum ($CDCl_3$, 400 MHz) of Compound **20.**

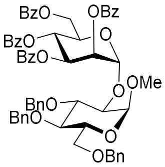

Methyl 2-*O*-(2,3,4,6-tetra-*O*-benzoyl-*α*-D-mannopyranosyl)-3,4,6-tri-*O*-benzyl-*α*-D-glucopyranoside (20)

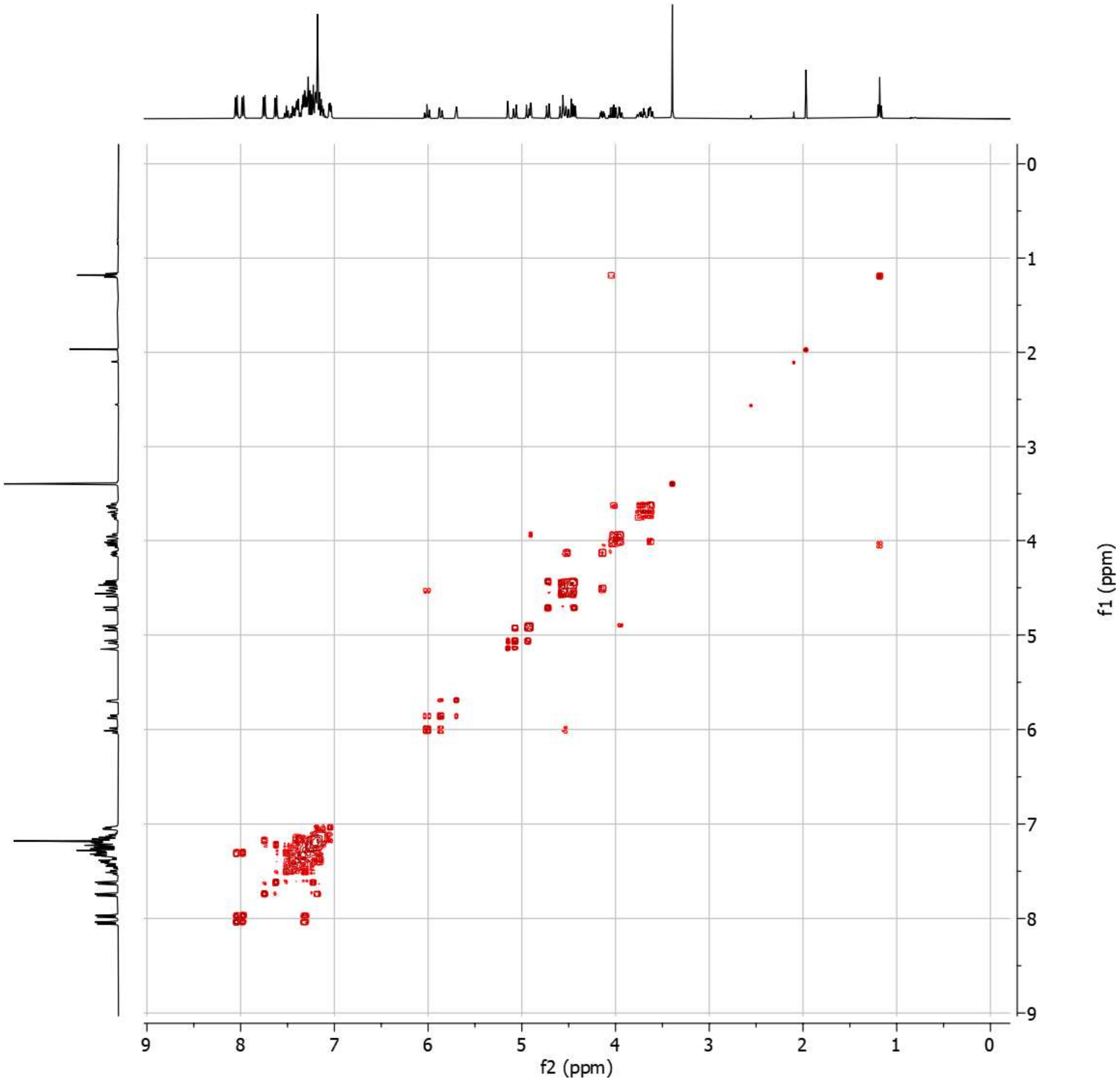

Figure S29. COSY NMR Spectrum ($CDCl_3$, 400 MHz) of Compound **20**.

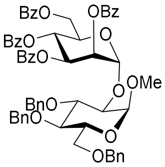

Methyl 2-*O*-(2,3,4,6-tetra-*O*-benzoyl-*α*-D-mannopyranosyl)-3,4,6-tri-*O*-benzyl-*α*-D-glucopyranoside (20)

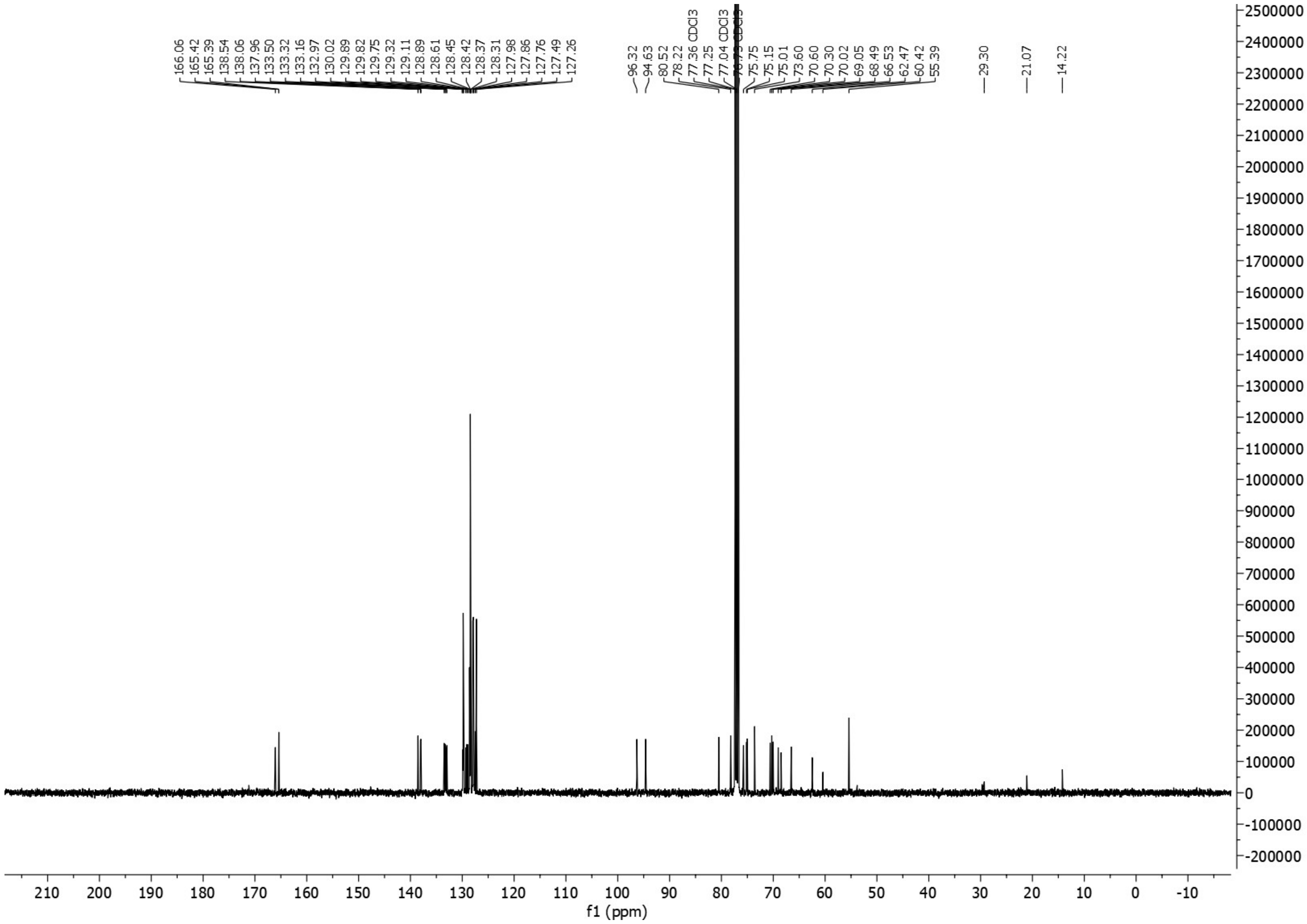

Figure S30. ^{13}C NMR Spectrum ($CDCl_3$, 101 MHz) of Compound **20**.

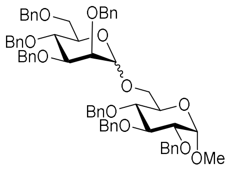

Methyl 2,3,4-tri-*O*-benzyl-6-*O*-(2,3,4,6-tetra-*O*-benzyl-α/β-D-mannopyranosyl)-α-D-glucopyranoside (22)

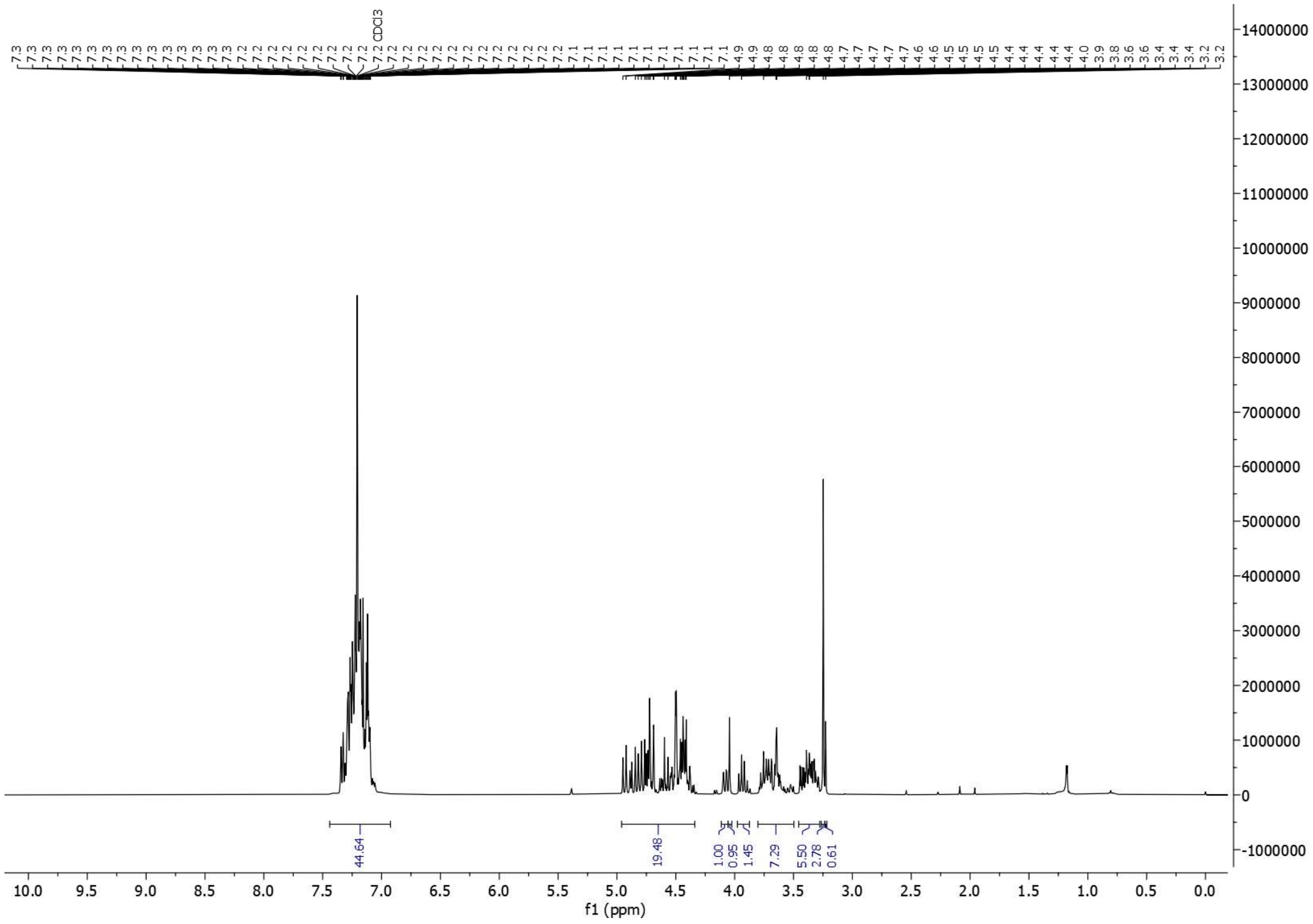

Figure S31. ^{1}H NMR Spectrum ($CDCl_3$, 400 MHz) of Compound **22.**

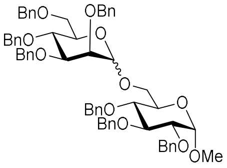

Methyl 2,3,4-tri-*O*-benzyl-6-*O*-(2,3,4,6-tetra-*O*-benzyl-α/β-D-mannopyranosyl)-α-D-glucopyranoside (22)

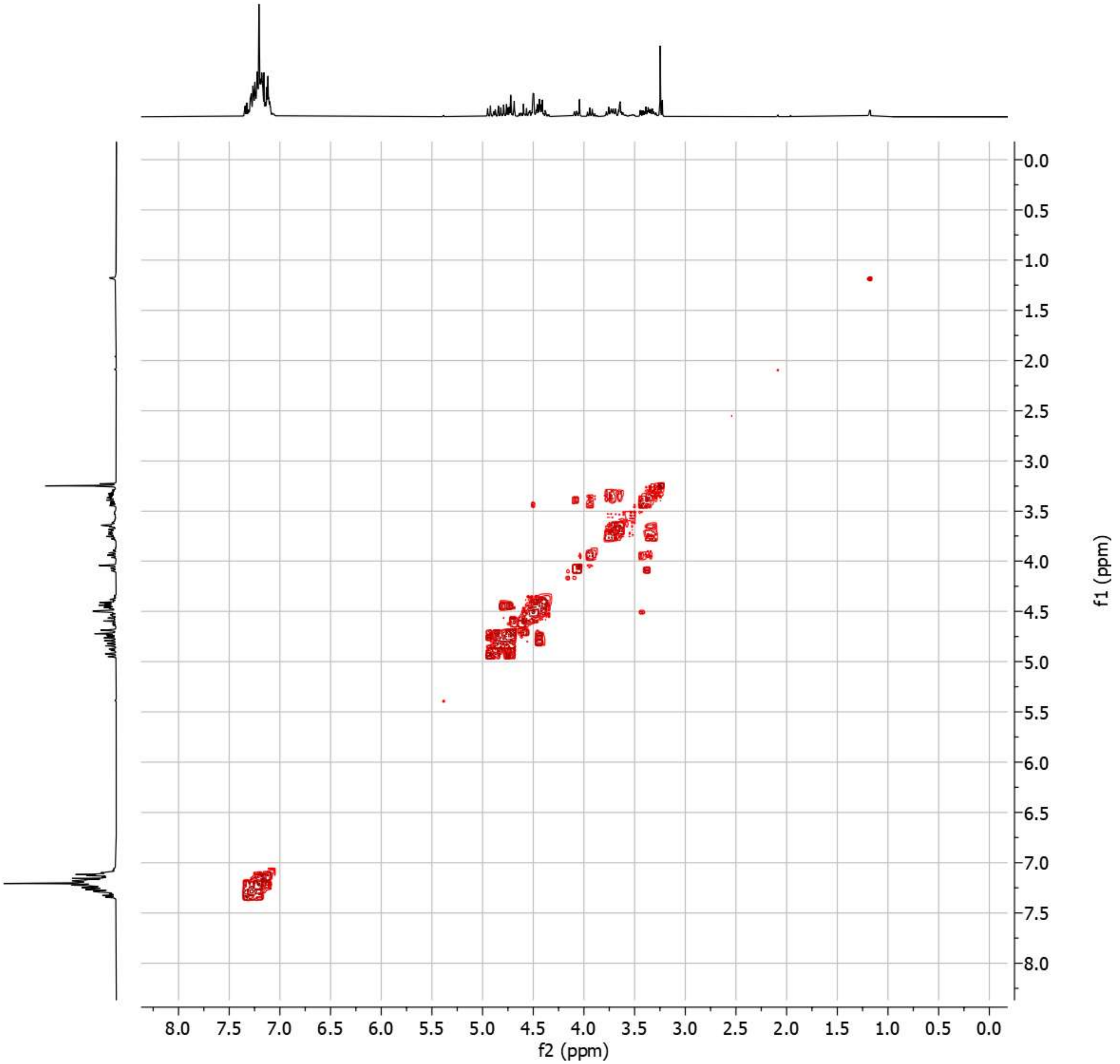

Figure S32. COSY NMR Spectrum ($CDCl_3$, 400 MHz) of Compound **22**.

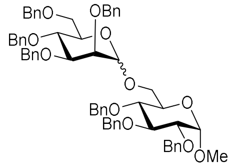

Methyl 2,3,4-tri-*O*-benzyl-6-*O*-(2,3,4,6-tetra-*O*-benzyl-α/β-D-mannopyranosyl)-α-D-glucopyranoside (22)

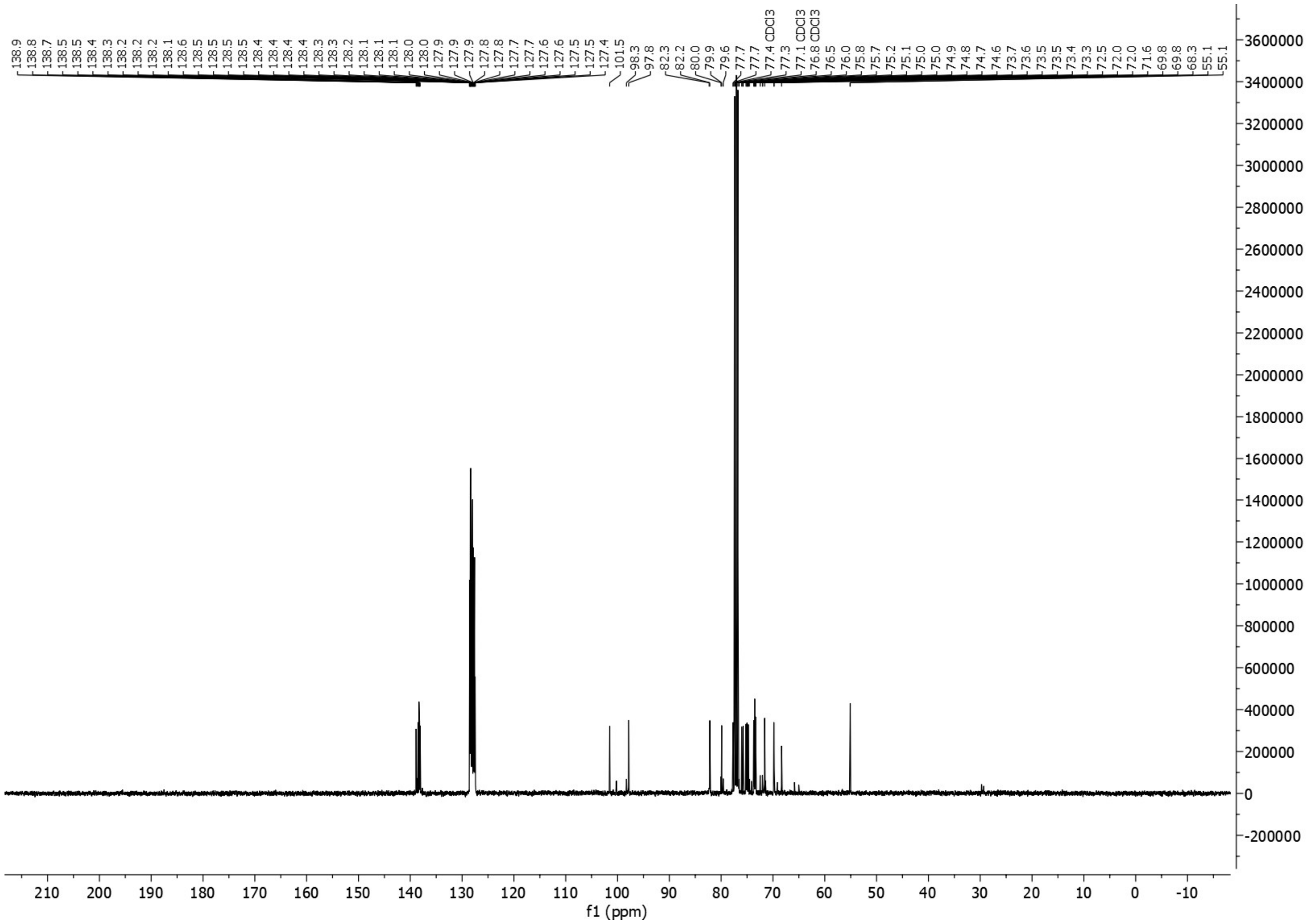

Figure S33. ^{13}C NMR Spectrum ($CDCl_3$, 101 MHz) of Compound **22**.

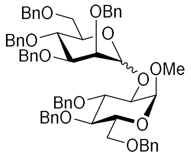

Methyl 3,4,6-tri-*O*-benzyl-2-*O*-(2,3,4,6-tetra-*O*-benzyl-α/β-D-mannopyranosyl)-α-D-glucopyranoside (23)

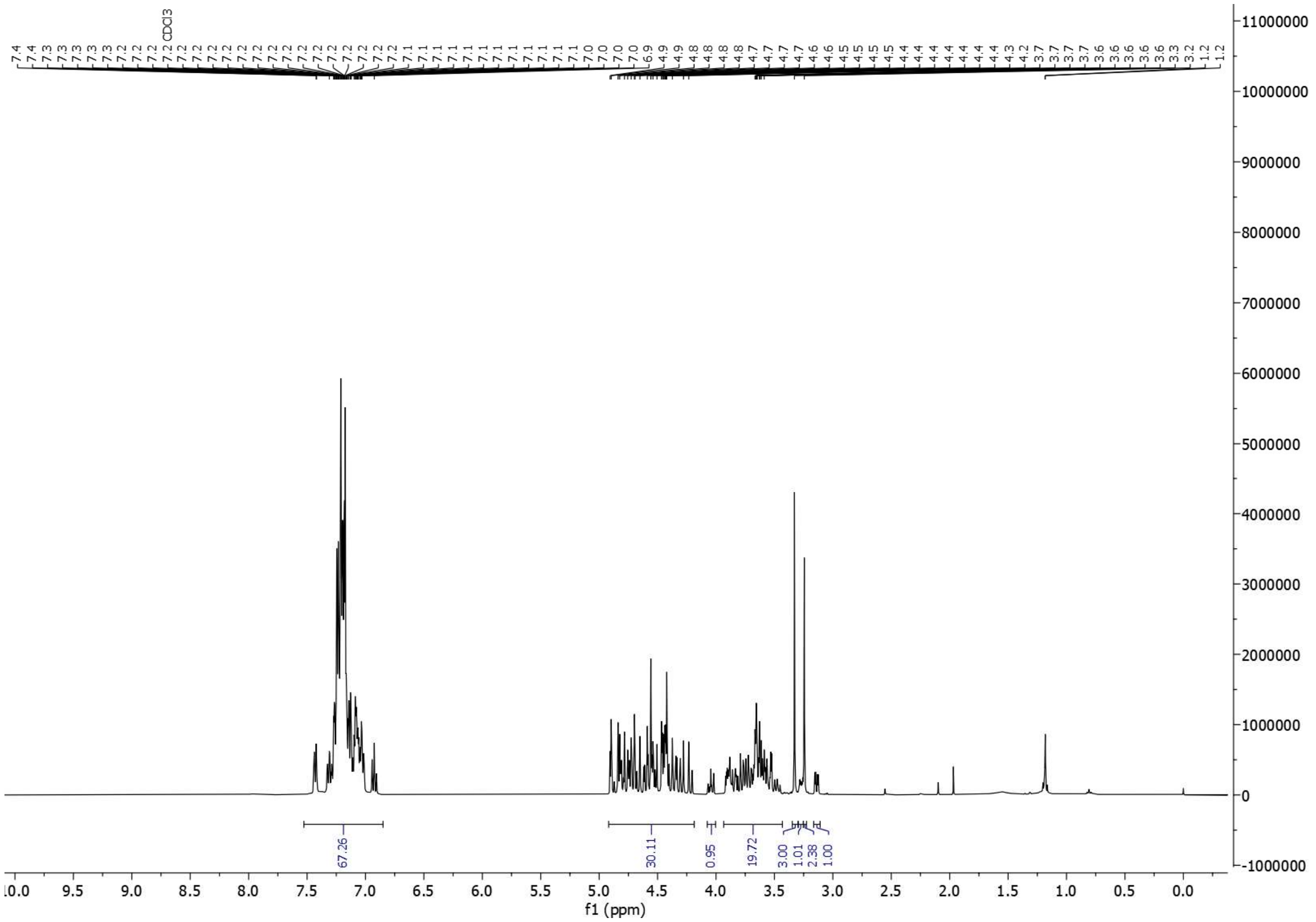

Figure S34. ^{1}H NMR Spectrum ($CDCl_3$, 400 MHz) of Compound **23.**

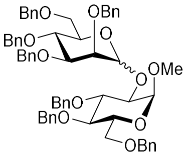

Methyl 3,4,6-tri-*O*-benzyl-2-*O*-(2,3,4,6-tetra-*O*-benzyl-*α*/β-D-mannopyranosyl)-*α*-D-glucopyranoside (23)

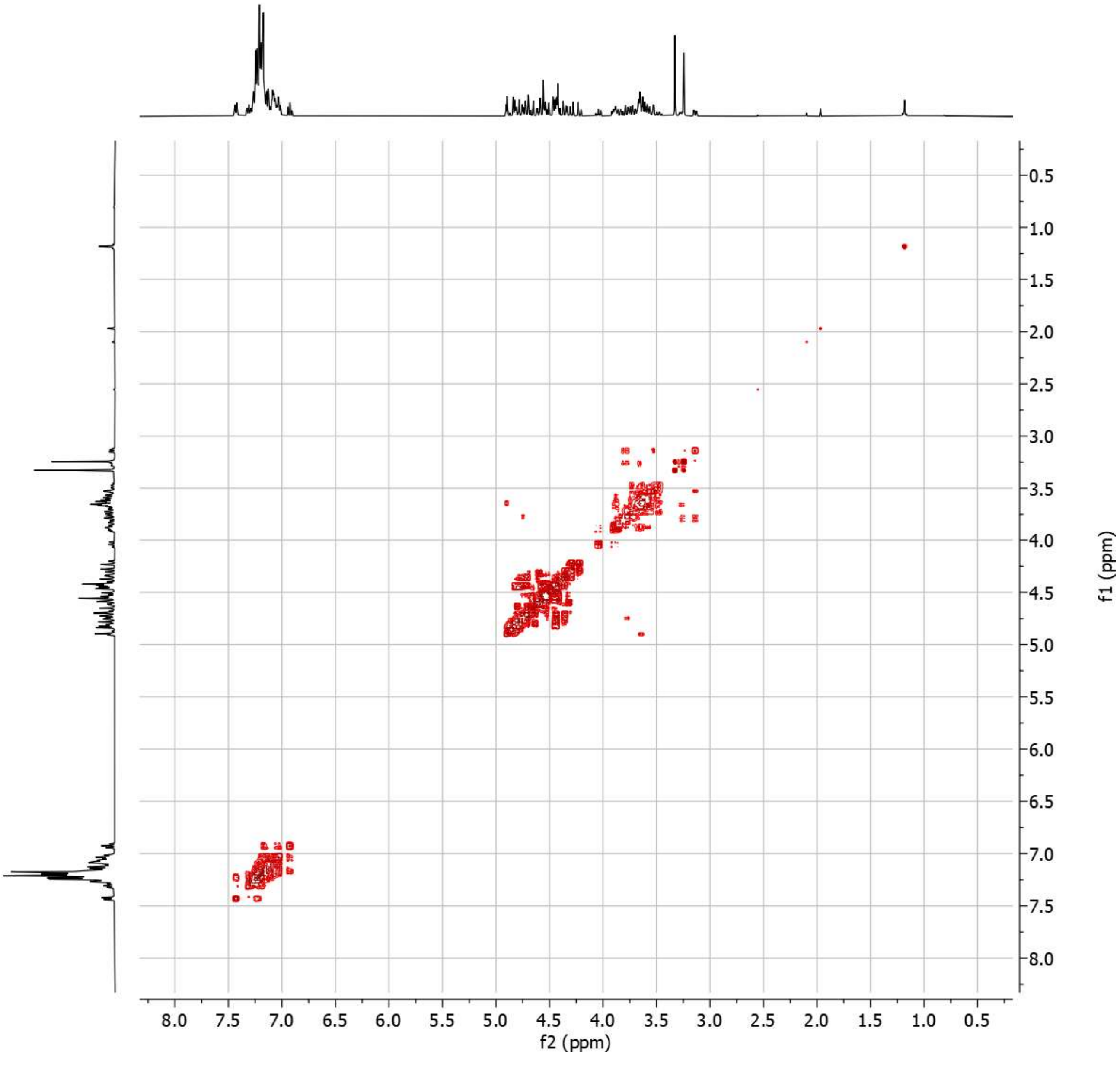

Figure S35. COSY NMR Spectrum ($CDCl_3$, 400 MHz) of Compound **23**.

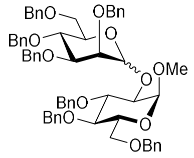

Methyl 3,4,6-tri-*O*-benzyl-2-*O*-(2,3,4,6-tetra-*O*-benzyl-α/β-D-mannopyranosyl)-α-D-glucopyranoside (23)

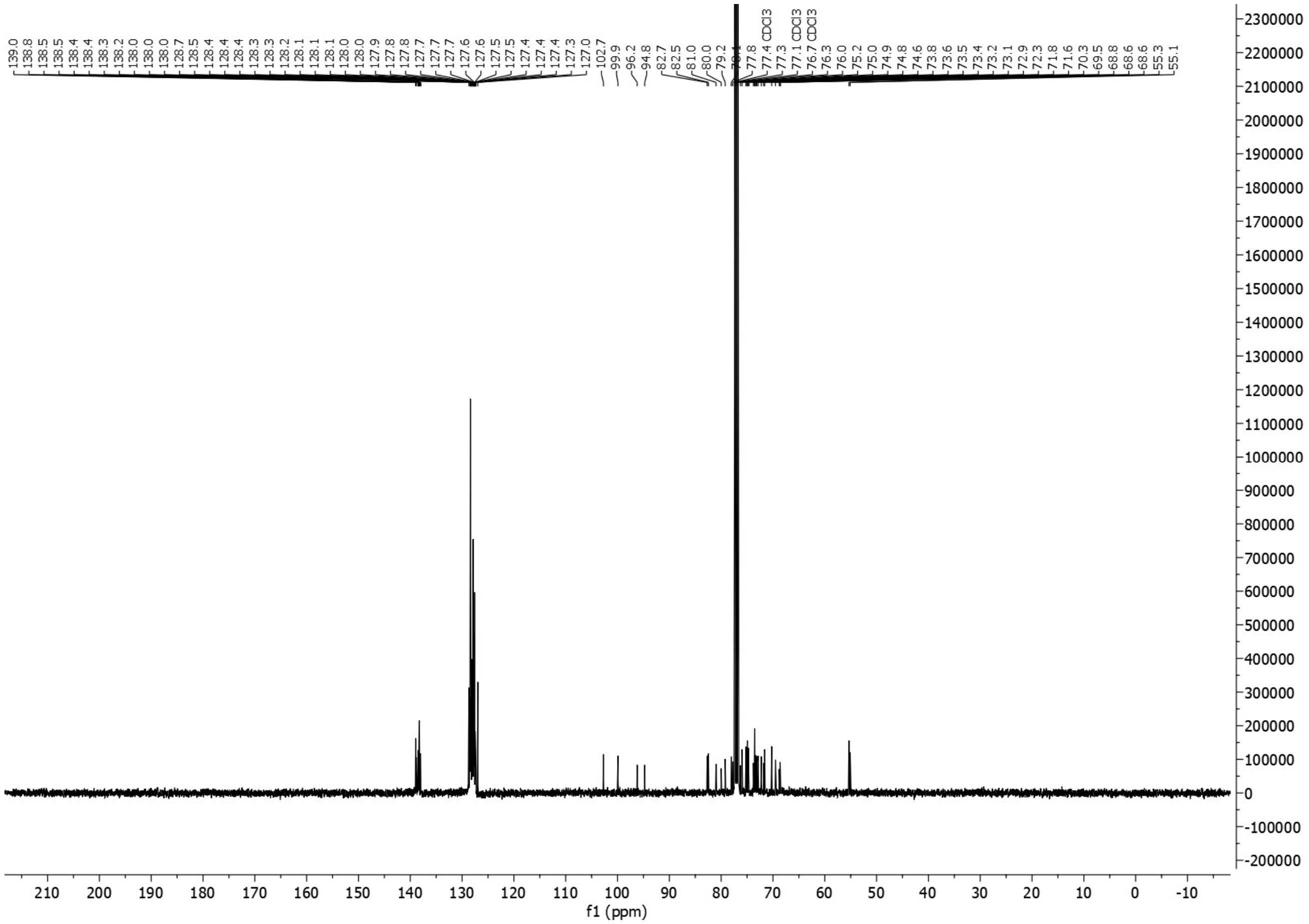

Figure S36. ^{13}C NMR Spectrum ($CDCl_3$, 101 MHz) of Compound **23**.

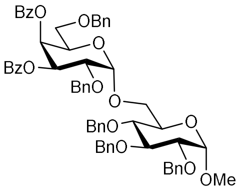

Methyl 6-*O*-(3,4-di-*O*-benzoyl-2,6-di-*O*-benzyl-β-D-galactopyranosyl)-2,3,4-tri-*O*-benzyl-*α*-D-glucopyranoside (25)

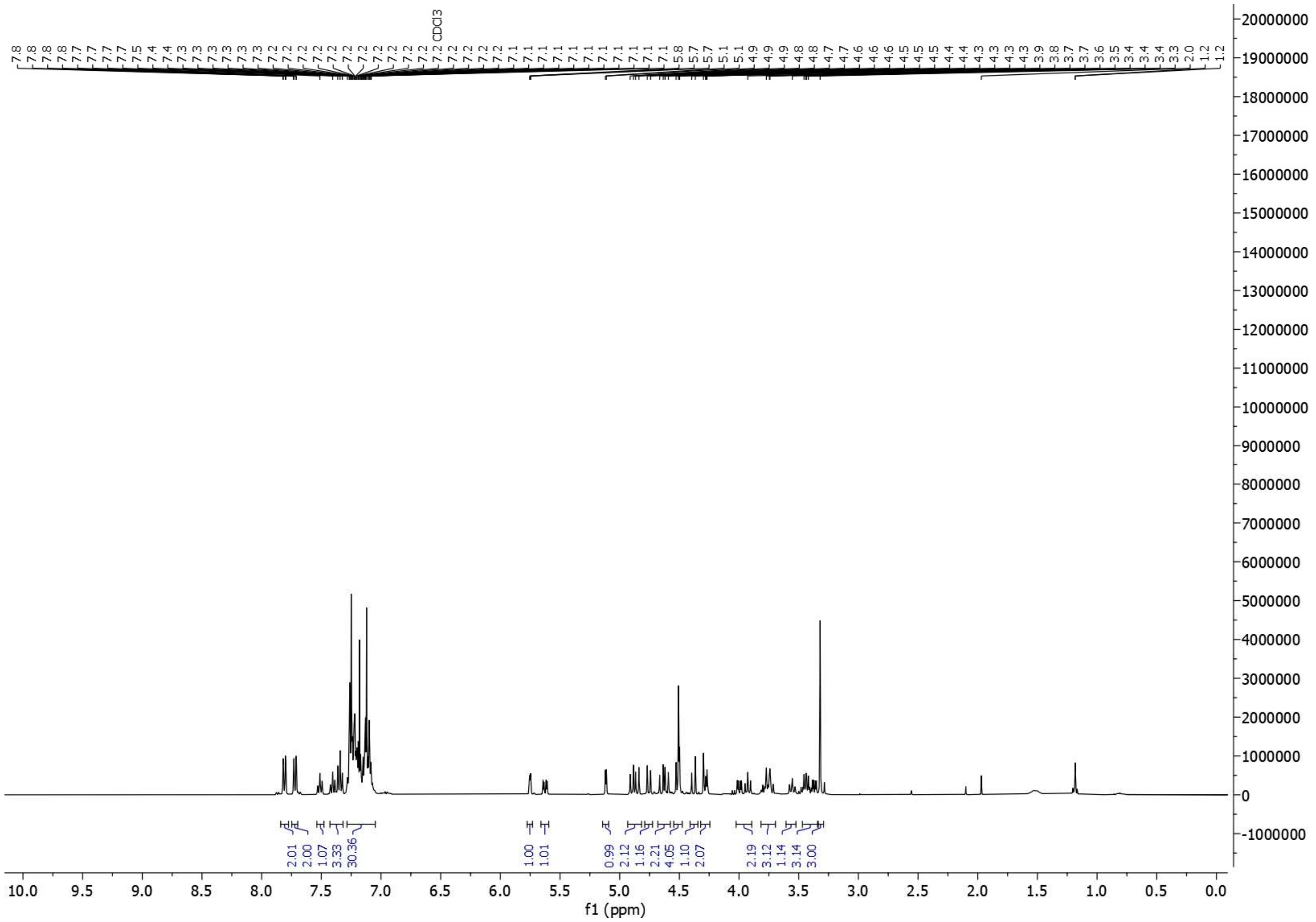

Figure S37. ^{1}H NMR Spectrum ($CDCl_3$, 400 MHz) of Compound **25.**

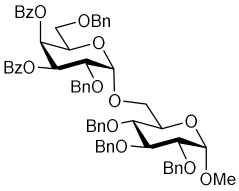

Methyl 6-*O*-(3,4-di-*O*-benzoyl-2,6-di-*O*-benzyl-β-D-galactopyranosyl)-2,3,4-tri-*O*-benzyl-α-D-glucopyranoside (25)

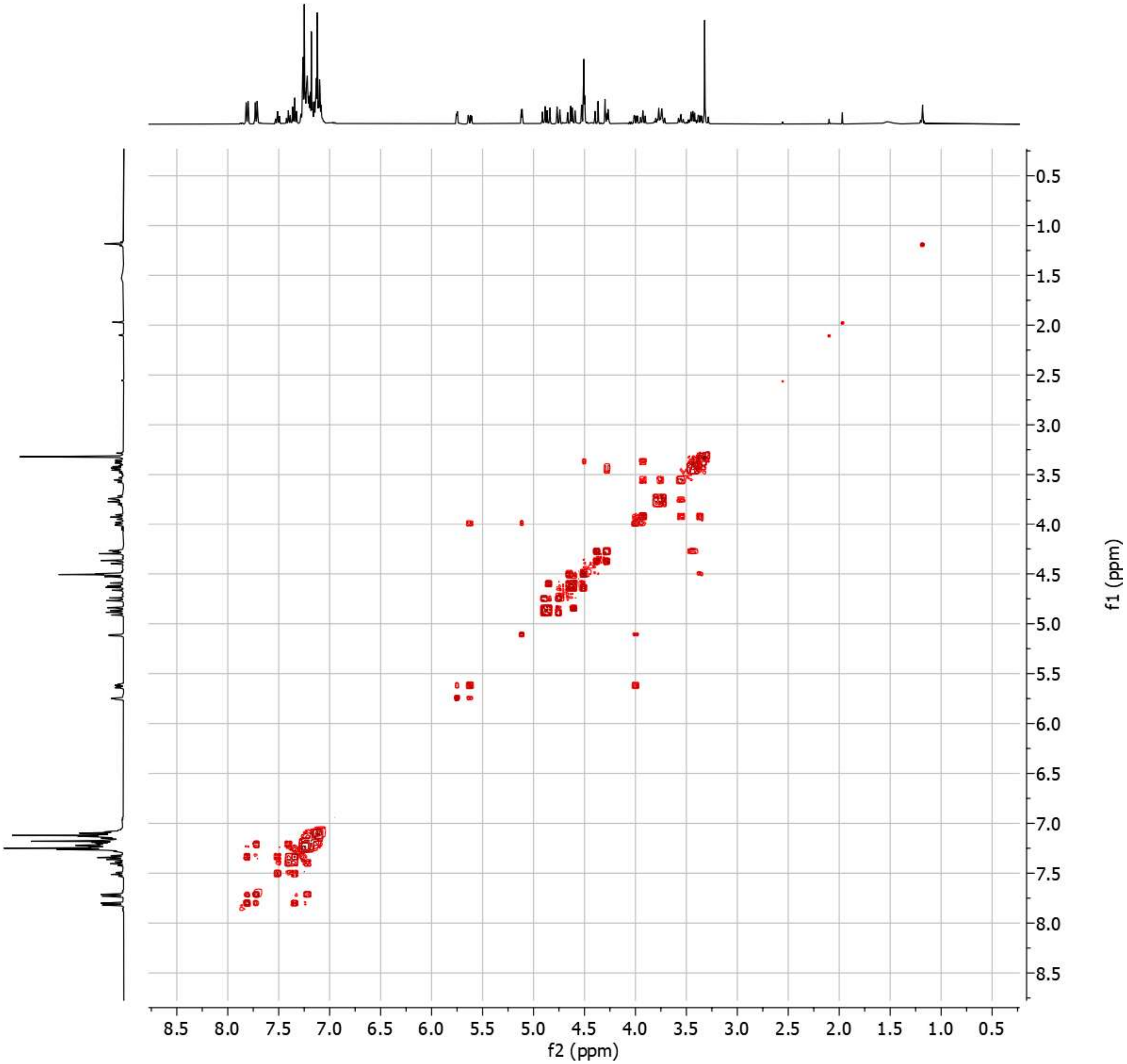

Figure S38. COSY NMR Spectrum ($CDCl_3$, 400 MHz) of Compound **25**.

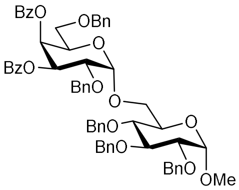

Methyl 6-*O*-(3,4-di-*O*-benzoyl-2,6-di-*O*-benzyl-β-D-galactopyranosyl)-2,3,4-tri-*O*-benzyl-*α*-D-glucopyranoside (25)

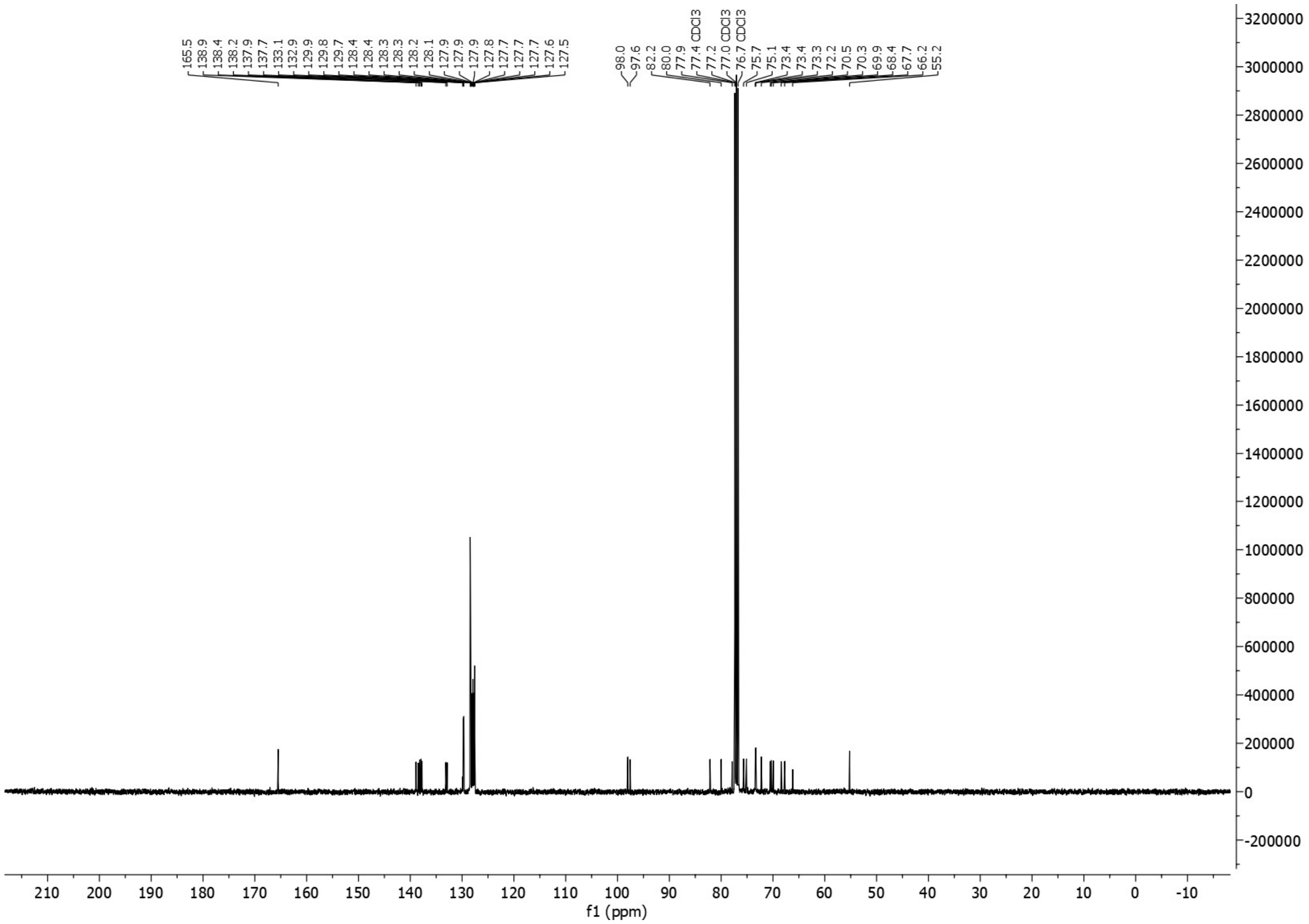

Figure S39. ^{13}C NMR Spectrum ($CDCl_3$, 101 MHz) of Compound **25**.

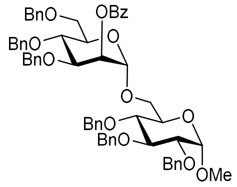

Methyl 6-*O*-(2-*O*-benzoyl-3,4,6-tri-*O*-benzyl-α-D-mannopyranosyl)-2,3,4-tri-*O*-benzyl-α-D-glucopyranoside (27)

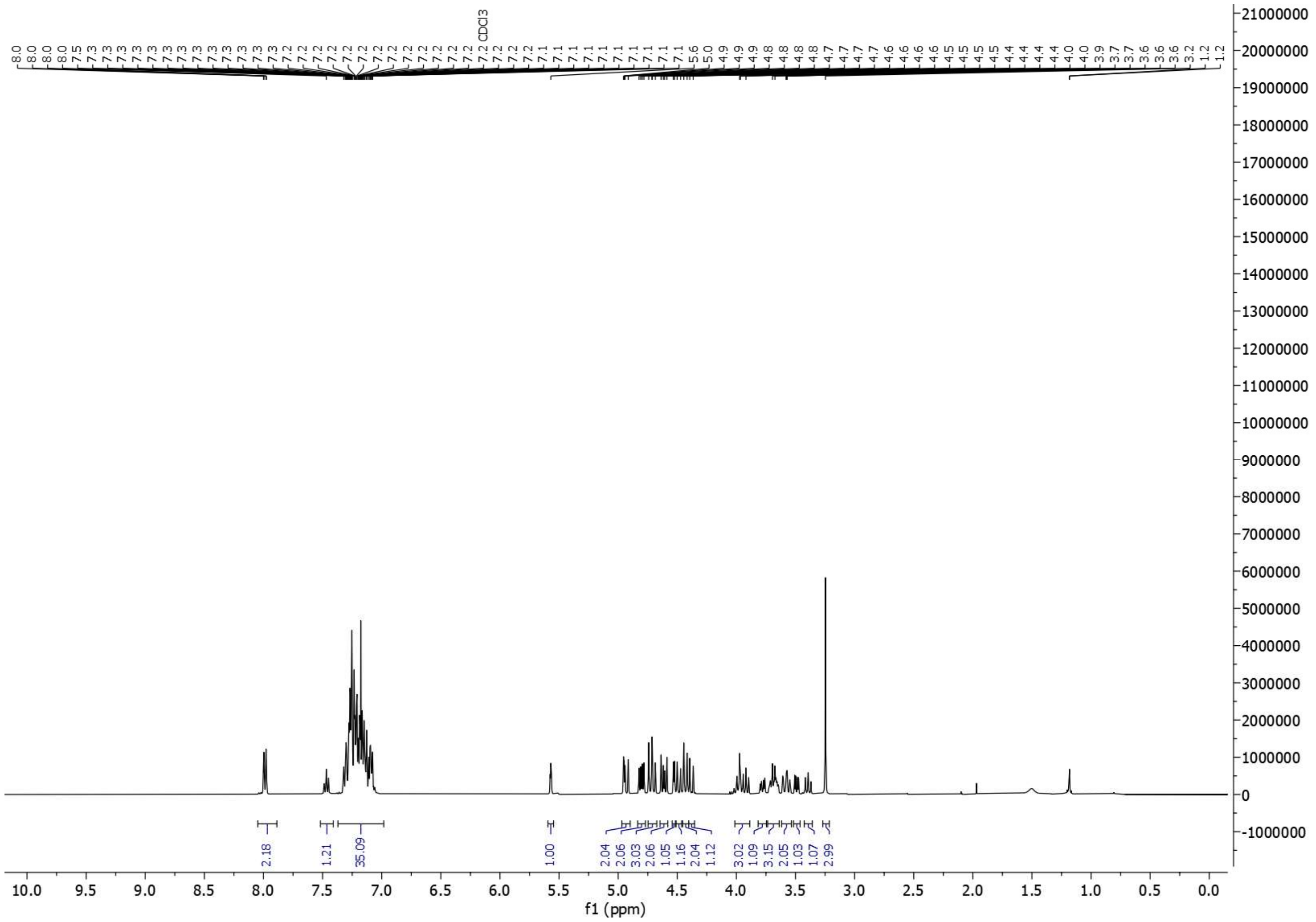

Figure S40. ^{1}H NMR Spectrum ($CDCl_3$, 400 MHz) of Compound **27.**

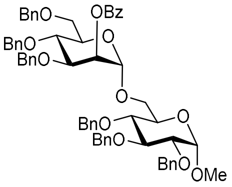

Methyl 6-*O*-(2-*O*-benzoyl-3,4,6-tri-*O*-benzyl-α-D-mannopyranosyl)-2,3,4-tri-*O*-benzyl-α-D-glucopyranoside (27)

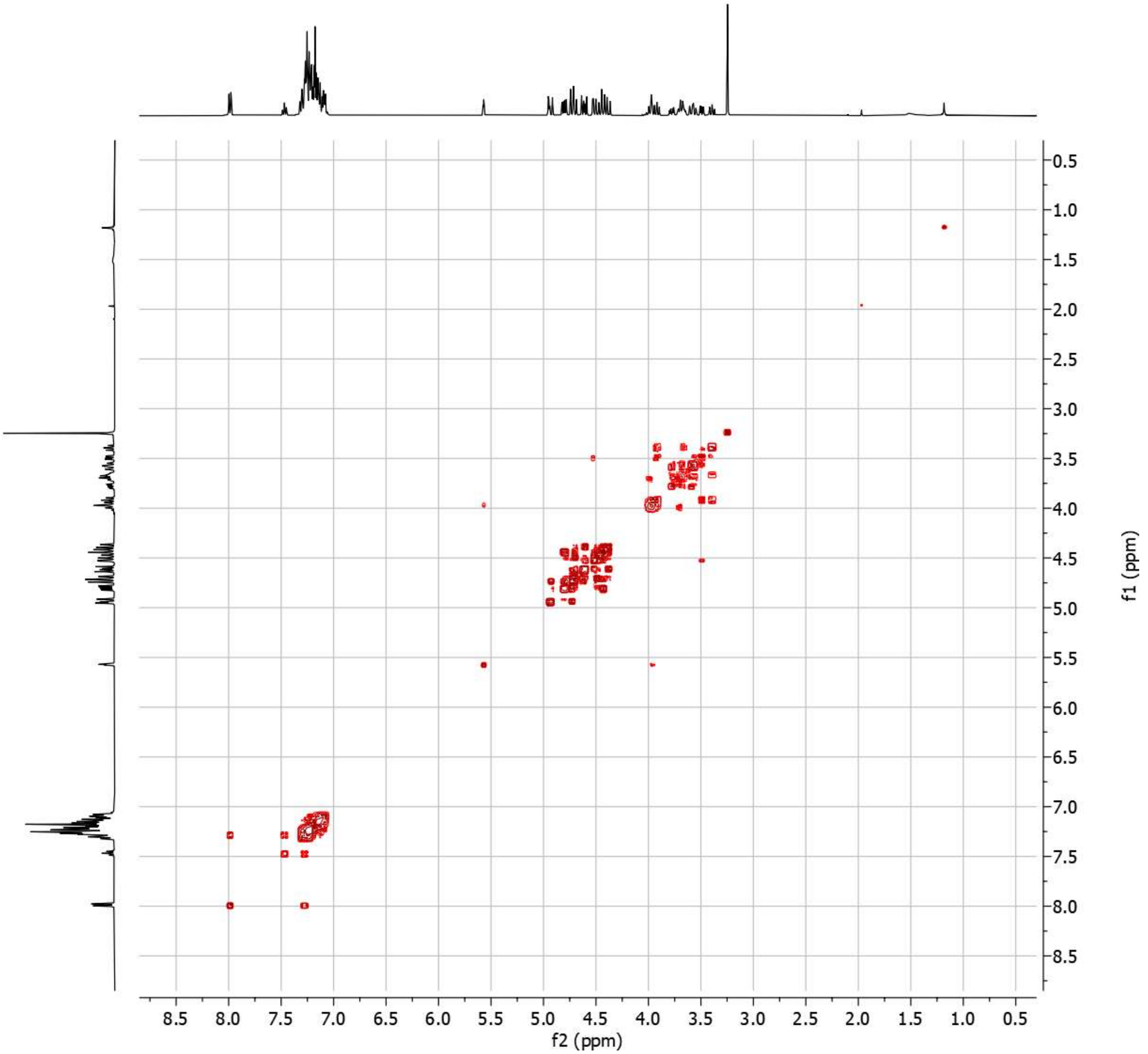

Figure S41. COSY NMR Spectrum ($CDCl_3$, 400 MHz) of Compound **27**.

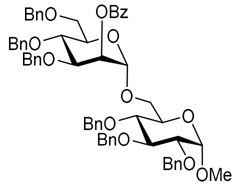

Methyl 6-*O*-(2-*O*-benzoyl-3,4,6-tri-*O*-benzyl-α-D-mannopyranosyl)-2,3,4-tri-*O*-benzyl-α-D-glucopyranoside (27)

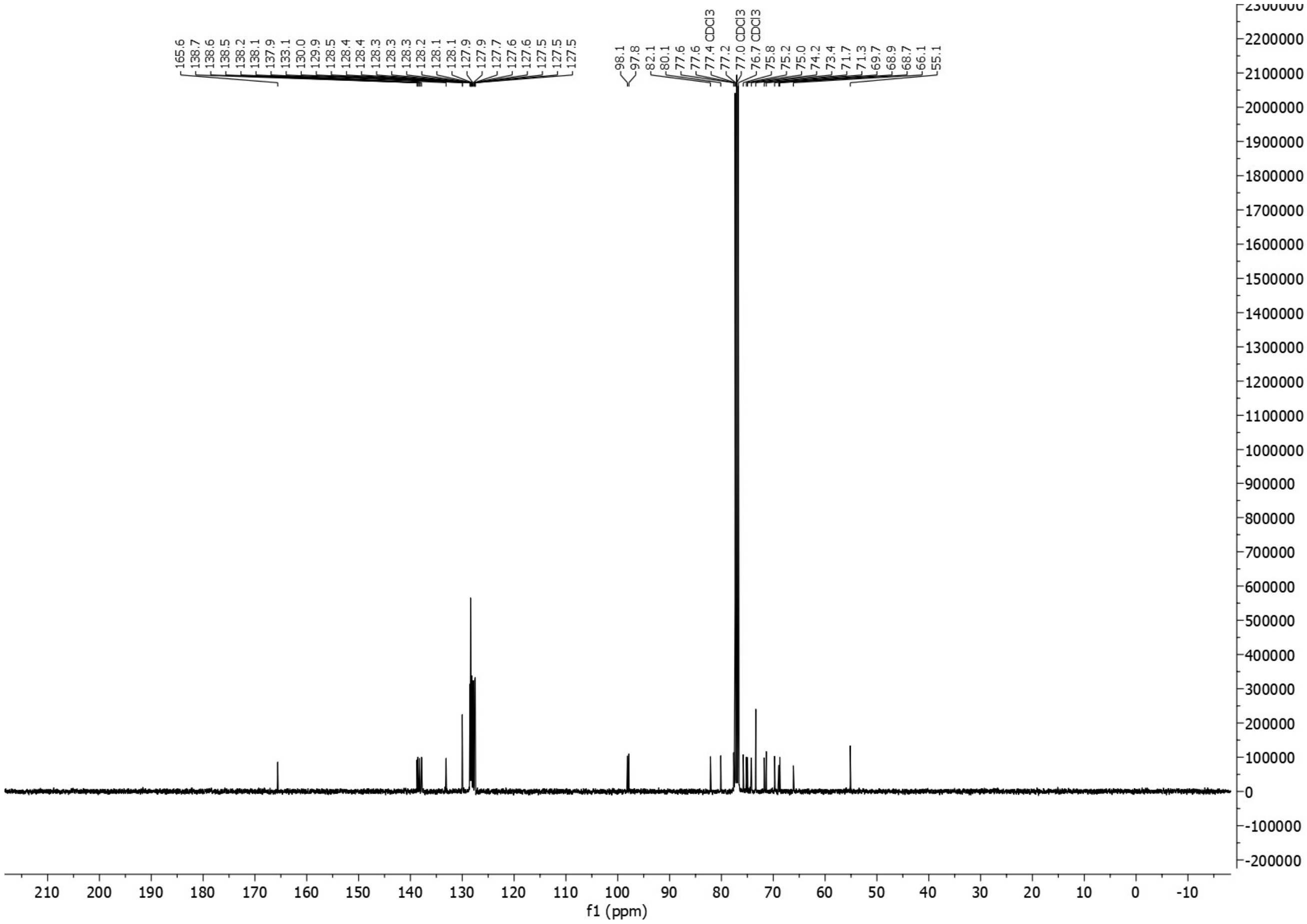

Figure S42. ^{13}C NMR Spectrum ($CDCl_3$, 101 MHz) of Compound **27**.
